# Sestrin2 Mitigates Neuronal Ferroptosis Following Subarachnoid Hemorrhage via Orchestration of the AMPK/PGC1α/Nrf2 Signaling Axis

**DOI:** 10.1002/cns.70908

**Published:** 2026-04-30

**Authors:** Yi Zhang, Xichen Wan, Hongyan Zhang, Zhaoyan Chen, Shining Xiao, Wansong Wang, Jun Wang, Renli Zheng, Xiuping Chen, Youqing Yang

**Affiliations:** ^1^ Rehabilitation Medicine Center, The First Affiliated Hospital, Jiangxi Medical College Nanchang University Nanchang Jiangxi China; ^2^ Department of Neurosurgery, The First Affiliated Hospital, Jiangxi Medical College Nanchang University Nanchang Jiangxi China; ^3^ Jiangxi University of Technology Nanchang Jiangxi China

**Keywords:** early brain injury, ferroptosis, oxidative stress, SESN2, subarachnoid hemorrhage

## Abstract

**Background:**

Early brain injury after subarachnoid hemorrhage (SAH) involves oxidative stress and ferroptosis. Sestrin2 (SESN2) regulates redox homeostasis, but its role in SAH‐induced ferroptosis remains unclear.

**Methods:**

SAH was induced by internal carotid puncture; HT22 cells were hemin‐treated. We assessed neurological deficits, brain edema, BBB integrity, iron levels, mitochondrial ultrastructure, and ferroptosis and signaling proteins by Western blot and immunofluorescence. SESN2 function was probed using knockdown, recombinant human SESN2 (rh‐SESN2), and pathway inhibitors.

**Results:**

rh‐SESN2 improved neurological outcomes, attenuated iron accumulation, and preserved mitochondrial morphology after SAH. In HT22 cells, rh‐SESN2 increased viability, decreased ROS and MDA, and partially restored SOD activity. rh‐SESN2 upregulated GPX4, SLC7A11, and Nrf2 and its nuclear translocation, whereas SESN2 knockdown exacerbated ferroptosis and reduced these proteins. Mechanistically, SESN2 activated AMPK and upregulated PGC1α, facilitating Nrf2 nuclear translocation and inducing antiferroptotic proteins. Pathway inhibition established an AMPK/PGC1α/Nrf2 hierarchy: AMPK inhibition suppressed PGC1α and Nrf2 activation, PGC1α inhibition reduced Nrf2 translocation, and ML385 abrogated SESN2's protective effects.

**Conclusion:**

SESN2 activates AMPK, which in turn upregulates PGC1α and promotes Nrf2 nuclear translocation, ultimately inhibiting neuronal ferroptosis after SAH. Targeting this AMPK/PGC1α/Nrf2 axis may provide a novel therapeutic approach for post‐SAH neuroprotection.

## Graphical abstract

The schematic summarizes SESN2's neuroprotective actions after subarachnoid hemorrhage. SESN2 activates an AMPK/PGC1α/Nrf2 signaling cascade that reduces oxidative stress, suppresses neuronal ferroptosis, and improves functional outcomes; these effects were validated by genetic knockdown and pharmacologic inhibition.

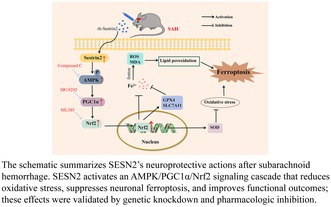

## 1 Introduction

Subarachnoid hemorrhage (SAH) represents a critical neurological emergency, accounting for 5%–10% of stroke cases, predominantly (85%) resulting from ruptured intracranial aneurysms [1, 2]. The condition carries a staggering 30‐day mortality approaching 50% [3, 4], with over one‐third of survivors experiencing complications that compromise functional recovery and life quality [3, 5, 6]. This significant global health burden necessitates novel therapeutic approaches [7, 8]. Despite technological improvements in surgical interventions and intensive care protocols, pharmaceutical options targeting underlying pathological mechanisms remain insufficient [9].

Early brain injury (EBI), developing within 72 h post‐SAH, critically determines prognosis through oxidative damage, compromise of the blood–brain barrier integrity, and neuronal death [10, 11]. Ferroptosis—a cell death pathway characterized by iron dependency and accumulation of lipid peroxides—as a key contributor to SAH‐induced neuronal damage [12, 13, 14]. In SAH, hemoglobin degradation products accumulate in the subarachnoid space, generating reactive oxygen species (ROS) that deplete antioxidants and propagate membrane rupture [15, 16]. The efficacy of iron chelators and ferroptosis inhibitors in SAH models confirms ferroptosis as a therapeutic target [17, 18].

Sestrin2 (SESN2), a stress‐inducible protein, regulates redox homeostasis and cellular adaptation [19]. While SESN2 modulates ferroptosis in ischemic stroke, liver diseases, and sepsis [20, 21, 22, 23, 24], its role in SAH‐induced neuronal ferroptosis remains unexplored. This represents a critical gap given SAH's unique iron pathophysiology, featuring massive subarachnoid hemoglobin degradation that directly drives neuronal lipid peroxidation [18]. AMP‐activated protein kinase (AMPK) maintains cellular homeostasis during stress [25], with SESN2 acting as its upstream activator [26]. AMPK enhances mitochondrial biogenesis and antioxidant capacity via peroxisome proliferator‐activated receptor gamma coactivator 1‐alpha (PGC1α) [27, 28]. Nuclear factor erythroid 2‐related factor 2 (Nrf2) neutralizes ROS and upregulates cytoprotective enzymes [29, 30], while PGC1α physically interacts with Nrf2 to amplify this antioxidant response [31, 32]. Emerging evidence reveals an integrated AMPK/PGC1α/Nrf2 axis wherein AMPK phosphorylates PGC1α to boost mitochondrial defenses while promoting Nrf2 nuclear translocation to suppress lipid peroxidation [33, 34]. Despite its protective roles in other diseases, this axis has not been investigated in SESN2‐mediated neuroprotection against ferroptosis in SAH.

We hypothesized that SESN2 attenuates post‐SAH neuronal ferroptosis by orchestrating the AMPK/PGC1α/Nrf2 signaling cascade, thereby preserving mitochondrial integrity and antioxidant capacity. This study aimed to elucidate the molecular processes involved in this pathway and assess its possible therapeutic implications in the context of brain damage following SAH.

## 2 Methods

### 2.1 Experimental Animals and Study Design

Adult male C57BL/6 mice (aged 8–10 weeks, weighing 22–25 g) were housed under controlled conditions with ethical approval from Nanchang University's Institutional Animal Care and Use Committee (CDYFY‐IACUC‐202309QR036). We chose male animals to minimize variability due to endogenous ovarian hormone fluctuations inherent to the female estrous cycle, which can influence cerebrovascular tone, inflammation, and molecular endpoints after subarachnoid hemorrhage (SAH) [35, 36]. For instance, estrogen ameliorates blood–brain barrier damage after experimental subarachnoid hemorrhage via the SHH pathway in male rats [37]. These sex‐related differences could confound the interpretation of our core research focus—the modulation of the Sestrin2‐Nrf2 pathway [38]. Animals were divided randomly into 8 groups with 6 animals per group: [1] Sham, [2] SAH, [3] SAH + Vehicle, [4] SAH + rh‐SESN2, [5] SAH + sh‐NC, [6] SAH + sh‐SESN2, [7] SAH + rh‐SESN2 + ML385, and [8] SAH + ML385.

### 2.2 SAH Model

We implemented the endovascular perforation technique with minor modifications [39]. Following anesthesia with 2% isoflurane, the right carotid arterial network was exposed. A specialized filament was advanced through the external carotid artery (ECA) into the internal carotid artery (ICA) until resistance was encountered, then further advanced 2–3 mm further to perforate the circle of Willis. Sham operation involved identical procedures without perforation. Specifically, animals in the Sham group received identical anesthesia, positioning, a neck incision, and exposure of the common, external, and internal carotid arteries. The surgical team simulated insertion of the filament up to the internal carotid artery but did not perform the final perforation of the circle of Willis; thus, no subarachnoid hemorrhage was induced in Sham animals. The duration of the procedure, wound closure, postoperative recovery, and monitoring were the same in Sham and SAH animals. Identical criteria for behavioral assessment and inclusion/exclusion were applied across groups. SAH severity was assessed using a standardized grading system [40], with mice scoring below 8 excluded from further analysis.

### 2.3 AAV‐Mediated SESN2 Knockdown and Pharmacological Intervention

For SESN2 knockdown, adeno‐associated viral vectors carrying either SESN2‐specific shRNA (AAV‐sh‐SESN2) or control shRNA (AAV‐sh‐NC) were obtained from GenePharma (Shanghai, China). Vector sequences appear in Table S1. We delivered 2 μL of viral preparation (1 × 10^12^ vg/mL) into the right lateral ventricle (coordinates: AP −0.4 mm, ML +1.0 mm, DV −2.2 mm from bregma) at 0.2 μL/min via microinfusion pump. The needle was maintained in position for 5 min following injection to ensure proper viral diffusion. Viral administration preceded SAH induction by 14 days to ensure optimal shRNA expression. Knockdown efficiency verification appears in Figure S1.

For pharmacological studies, rh‐SESN2 (3 μg/mouse, Sigma‐Aldrich) was administered intracerebroventricularly 2 h after SAH induction [38, 41]. The in vivo rh‐SESN2 dosing regimen used in this study (3 μg/mouse, ICV) was chosen based on our prior dose‐finding experiments in the same SAH model where rh‐SESN2 was tested at 1, 3, and 9 μg/mouse; 3 μg/mouse produced robust and reproducible neuroprotective effects while higher doses did not yield additional benefit [38]. The in vitro rh‐SESN2 concentrations for HT22 cells were selected based on published HT22 ischemia studies and were confirmed by pilot toxicity assays showing no cytotoxicity at the employed concentrations [41]. ML385, a specific Nrf2 inhibitor (30 mg/kg) [38, 42], was delivered intraperitoneally 2 h before SAH to evaluate Nrf2 pathway involvement in SESN2‐mediated protection. Control groups received equivalent volumes of vehicle (PBS) or AAV‐sh‐NC following identical administration protocols.

### 2.4 Neurological Assessment

Neurological impairments were assessed 24 h post‐SAH using a balance beam protocol by an investigator blinded to experimental groupings. This evaluation assessed each mouse's ability to maintain equilibrium on a narrow beam (1‐cm width) for 60 s. Performance was scored on a scale from 0 (falling within 20 s) to 4 (traversing the beam with no foot slip).

### 2.5 Morris Water Maze (MWM) Test

Spatial learning and memory were assessed using a circular Morris water maze [43]. The pool (diameter ≈ 150 cm) was filled with water maintained at 22°C–25°C and rendered opaque with nontoxic white paint. A circular escape platform (diameter ≈ 10 cm) was submerged 1 cm below the water surface and kept at a constant location within one quadrant during acquisition. Distinct extra‐maze visual cues were mounted on the walls of the testing room and remained unchanged throughout the experiment.

The MWM protocol comprised two components: place navigation (acquisition) and spatial probe (memory retention). Place navigation was performed on postoperative days 7–10. Training occurred at a consistent time each day, with four training trials per day. For each trial, mice were released from one of two pseudorandomized entry points and gently placed into the water facing the pool wall; the trial ended when the animal reached the platform or when the maximum trial duration of 60 s elapsed. Escape latency (time from release to platform) and swim path were recorded for each trial. The spatial probe test was conducted 24 h after the final acquisition session. The platform was removed, and mice were released from a start point opposite the former platform location and allowed to swim for 60 s. During the probe, primary endpoints included the number of crossings over the former platform location. All testing and analyses were performed by investigators blinded to group assignment. Data from individual trials and group means (mean ± SD) were analyzed as described in the Statistical Analysis section.

### 2.6 Brain Water Content Assessment

Cerebral edema was quantified 24 h post‐SAH. Following euthanasia, researchers quickly harvested and separated each brain into left and right hemispheres. Brain segments were immediately weighed to obtain wet weights, then oven‐dried at 105°C for 24 h before measuring dry weights. The percentage of brain water content was then calculated using the equation: [(wet weight−dry weight)/wet weight] × 100%.

### 2.7 Iron Content Measurement

Tissue iron concentration was quantified using a colorimetric assay kit (Sigma‐Aldrich). Brain samples underwent acid homogenization followed by centrifugation. The resulting supernatant was incubated with iron detection reagent at room temperature (RT), with absorbance measured at 593 nm. The iron content was expressed as mg/g protein.

### 2.8 Transmission Electron Microscopy

Ipsilateral cortical tissue samples (1 mm^3^) underwent primary fixation in 2.5% glutaraldehyde (2 h), followed by secondary fixation in 1% osmium tetroxide. After dehydration through ascending ethanol concentrations, samples were embedded in epoxy resin. Ultrathin sections (70 nm) were prepared, enhanced with uranyl acetate and lead citrate stains, and visualized using transmission electron microscopy for ultrastructural analysis (HT7700, Hitachi, Tokyo, Japan). Mitochondrial morphology and density were assessed by a researcher who was unaware of the treatment group.

### 2.9 Cell Culture and In Vitro SAH Model

HT22 cells were maintained in high‐glucose DMEM supplemented with 10% fetal bovine serum and 1% penicillin/streptomycin mixture. The incubation conditions were set at 37°C with a 5% CO_2_ atmosphere. The cells were subcultured every 3–4 days when the cell density reached approximately 80%–90% confluence. For the in vitro SAH model, hemin (MCE, China) was dissolved in 0.1 M NaOH, and HT22 cells were exposed to complete medium containing hemin (200 μM) for 24 h to mimic SAH conditions [44].

### 2.10 Cell Transfection and Treatment

Cells were plated at a density of 2 × 10^5^ cells per well in 6‐well plates and allowed to adhere for 24 h before experimentation. Transfections employed Lipofectamine 2000 (Invitrogen) according to the manufacturer's protocols. Three SESN2‐targeting siRNAs and a negative control sequence were designed based on murine SESN2 mRNA (NM_144907; sequences in Table S2). Cells were harvested 72 h posttransfection, with knockdown efficiency verified by qRT‐PCR and Western blotting (Figure S2). The most effective sequence, si‐M‐SESN2‐2, was selected for subsequent experiments.

To examine signaling pathway involvement, cells were pretreated with specific inhibitors for 2 h: Compound C (10 μM, AMPK inhibitor) [45], SR18292 (20 μM, PGC1α inhibitor) [46], or ML‐385 (5 μM) [18], followed by rh‐SESN2 treatment (12.5 ng/mL) [36] for an additional 2 h prior to hemin exposure. For rescue experiments, HT22 cells were treated as indicated; Compound C was used to inhibit AMPK and sulforaphane (SFN, 10 μM) was used to pharmacologically activate Nrf2. ACSL4 detection and Ferrostatin‐1 control experiments. HT22 cells were used to assess ACSL4 expression and to evaluate Ferrostatin‐1 (Fer‐1) as a ferroptosis inhibitor control. rh‐SESN2 and Fer‐1 were applied according to optimized pre‐experiment conditions (rh‐SESN2 12.5 ng/mL; Fer‐1 1 μM), with pretreatment given 2 h prior to Hemin exposure.

### 2.11 Cell Viability Assay

A Cell Counting Kit‐8 (MCE, USA) was used to evaluate cell viability following the manufacturer's guidelines. In brief, HT22 cells were placed in 96‐well plates and exposed to the specified treatments. After treatment, wells were supplemented with CCK‐8 reagent and incubated at 37°C for 2 h. After the incubation, the absorbance at 450 nm was measured using a microplate reader. The viability results were normalized and expressed as percentages relative to the sham group.

### 2.12 Oxidative Stress Analysis

Oxidative parameters were assessed in tissue homogenates and cell lysates prepared in PBS. After centrifugation (12,000 rpm, 10 min, 4°C), supernatants underwent analysis. Malondialdehyde (MDA) content in HT22 cells was quantified using a commercial detection kit (Beyotime), with absorbance measured at 532 nm and results expressed as nmol/mg protein. Superoxide dismutase (SOD) activity in both tissue and cell extracts was determined using a SOD kit (Beyotime Biotechnology). Samples were incubated with assay reagents (37°C, 30 min), followed by spectrophotometric reading at 450 nm, with activity expressed as U/mg protein. For intracellular ROS detection, HT22 cells were incubated with DCFH‐DA (10 μM, MCE) at 37°C for 30 min. Fluorescent signals were detected using excitation at 488 nm and emission was measured at 525 nm wavelength with data presented as relative fluorescence units per mg protein, normalized to sham controls. All measurements were performed in triplicate.

### 2.13 Western Blot Analysis

Proteins were isolated from brain tissues or HT22 cells using RIPA lysis buffer (Beyotime) supplemented with Roche protease and phosphatase inhibitor cocktails. We quantified protein concentrations using the BCA method (Thermo Scientific). Samples (30 μg per lane) were resolved on 10%–15% SDS‐polyacrylamide gels and subsequently transferred to PVDF membranes (Millipore). Membranes were blocked in 5% nonfat milk in TBST (1 h, RT) and incubated overnight (4°C) with primary antibodies in 5% milk or BSA: rabbit anti‐ZO‐1 (1:2000, Abcam, ab307799), rabbit anti‐occludin (1:1000, Abcam, ab216327), rabbit anti‐Sesn2 (1:1000, Abcam, ab178518), mouse anti‐GPX4 (1:2000, Abcam, ab125066), rabbit anti‐SLC7A11 (1:1000, Abcam, ab175186), rabbit anti‐phospho‐AMPKα (1:1000, Cell Signaling Technology, #2535), rabbit anti‐AMPKα (1:2000, Cell Signaling Technology, #2532), mouse anti‐PGC1α (1:3000, Abcam, ab176328), rabbit anti‐Nrf2 (1:1000, Cell Signaling Technology, #12721), rabbit anti‐β‐actin (1:10,000, Abcam, ab179467), rabbit anti‐Histone H3 (1:5000, Cell Signaling Technology, #9717), ACSL4 (1:2000, 22401‐1‐AP). Following the washing procedure, the membranes were treated with horseradish peroxidase‐linked secondary antibodies (1:5000, Cell Signaling Technology) for 90 min at RT.

### 2.14 Immunofluorescence Staining

For tissue analysis, mice were anesthetized and perfused with cold PBS followed by 4% paraformaldehyde. Brains were postfixed (24 h), dehydrated in 30% sucrose, and cryosectioned (10 μm). For cellular analysis, HT22 cells on coverslips underwent fixation using 4% paraformaldehyde (15 min, RT). All samples were permeabilized with 0.3% Triton X‐100 (15 min) and blocked with 10% normal goat serum (1 h, RT). Samples were incubated overnight (4°C) with primary antibodies: mouse anti‐NeuN (1:200, Abcam, ab104224), mouse anti‐GPX4 (1:200, Abcam, ab125066), rabbit anti‐SLC7A11 (1:200, Abcam, ab307601), and rabbit anti‐Nrf2 (1:200, Abcam, ab62352). After PBS washing, samples underwent incubation with Alexa Fluor‐linked secondary antibodies (1:500, Invitrogen, 1 h, RT) and counterstained with DAPI (1:1000, Sigma‐Aldrich). Visualization were captured using an Olympus FV3000 confocal system, and fluorescence intensity was quantified using ImageJ software.

### 2.15 Statistical Analysis

Data are presented as mean ± standard deviation (mean ± SD), and the number of biological replicates is indicated in each figure legend. Statistical analyses were performed using GraphPad Prism 9.0. Normality was assessed by the Shapiro–Wilk test and homogeneity of variance by Levene's test. For datasets meeting normality and homogeneity assumptions, two‐group comparisons were performed with two‐tailed unpaired Student's *t*‐tests; multiple group comparisons were analyzed by one‐way ANOVA followed by Tukey's post hoc test. When assumptions were violated, nonparametric alternatives were used (Mann–Whitney *U* test for two‐group comparisons; Kruskal‐Wallis test with Dunn's post hoc for multiple comparisons). Repeated‐measures or time‐course data were analyzed by two‐way ANOVA or repeated‐measures ANOVA with appropriate post hoc tests. All tests were two‐tailed and significance was set at *p* < 0.05. No data points were excluded unless pre‐specified technical/experimental failures were documented.

## 3 Results

### 3.1 SESN2 Improves Spatial Memory and Learning Deficits, Neurobehavioral Function, Reduces Brain Edema, and Decreases Neuronal Ferroptosis After SAH

**FIGURE 1:**
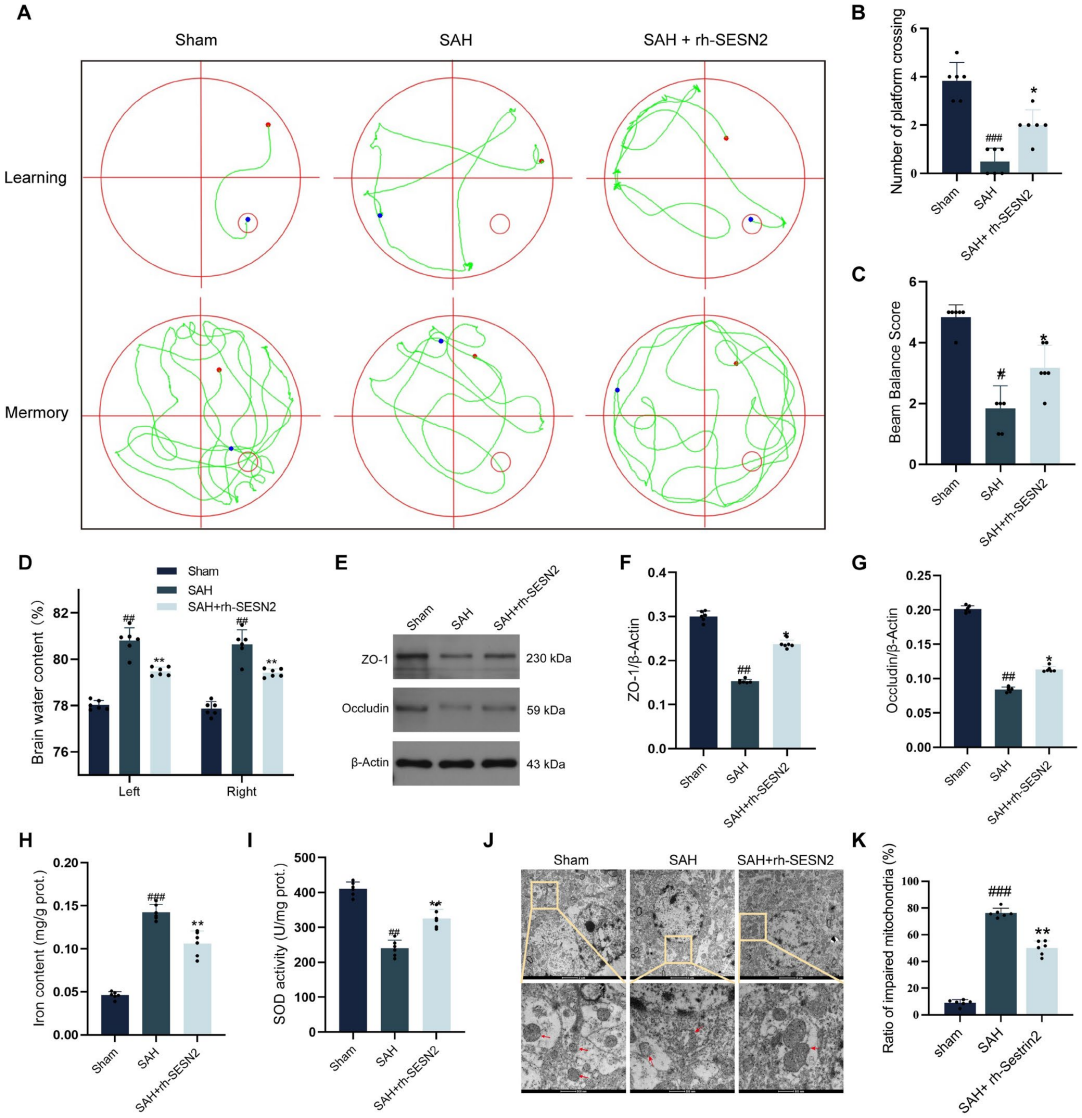
SESN2 treatment ameliorates brain injury and blood–brain barrier (BBB) disruption following SAH. (A, B) The Morris water maze was used to assess spatial memory and learning abilities. (C) Beam balance test scores showing neurological function across groups. (D) Brain water content in left and right hemispheres after SAH. (E) Western blot analysis and quantification of BBB integrity markers ZO‐1 (F) and Occludin (G). (H) Quantification of brain tissue iron content. (I) SOD activity in brain tissue. (J, K) Transmission electron microscopy images showing mitochondrial morphology in cortical neurons, Upper panels (scale bar = 2 μm) show cellular fields; lower panels (scale bar = 500 nm) show magnified views of highlighted areas; Red arrows indicate mitochondrial damage characterized by swelling, cristae disruption, and outer membrane rupture, which is most severe in the SAH group and partially attenuated in the rh‐SESN2 treatment group. Data indicated as mean ± SD. *n* = 6 per group. #*p* < 0.05, ##*p* < 0.01, ###*p* < 0.001 vs. Sham; *p < 0.05, **p < 0.01 vs. SAH.

Our previous research demonstrated that SESN2 protein levels in the ipsilateral cortex significantly increased following SAH, peaking at 24 h postinjury [36]. To define the cellular source of SESN2 after SAH, we refer to our previously published analysis in the same SAH model [38]. In that study, double immunofluorescence staining (SESN2/NeuN and SESN2/Iba1) revealed SESN2 expression in both neurons and microglia, with predominant neuronal localization and marked upregulation in neurons after SAH. Accordingly, this study focuses primarily on neuronal ferroptosis and the role of SESN2 in neuronal antioxidant responses. Building on these observations, we treated SAH mice with rh‐SESN2. Compared with vehicle‐treated SAH animals, rh‐SESN2 markedly improved spatial learning and memory in the Morris water maze (Figure 1A,B), enhanced neurobehavioral performance on the beam‐balance test (Figure 1C), and reduced cerebral edema as indicated by decreased brain water content in both hemispheres (Figure 1D). Western blot analysis revealed that SAH induced a marked decrease in tight junction proteins (ZO‐1 and occludin) in the ipsilateral cortex, which was partially restored by rh‐SESN2 treatment (Figure 1E–G). Furthermore, rh‐SESN2 significantly reduced post‐SAH iron accumulation (Figure 1H) and improved SOD activity (Figure 1I), suggesting enhanced antioxidant capacity. Transmission electron microscopy demonstrated that rh‐SESN2 treatment notably attenuated SAH‐induced mitochondrial damage in cortical neurons, characterized by reduced swelling, preserved cristae, and intact outer membranes (Figure 1J,K).

### 3.2 SESN2 Reduces Oxidative Stress and Ferroptosis After SAH by Activating the Nrf2 Signaling Pathway in Mice

**FIGURE 2:**
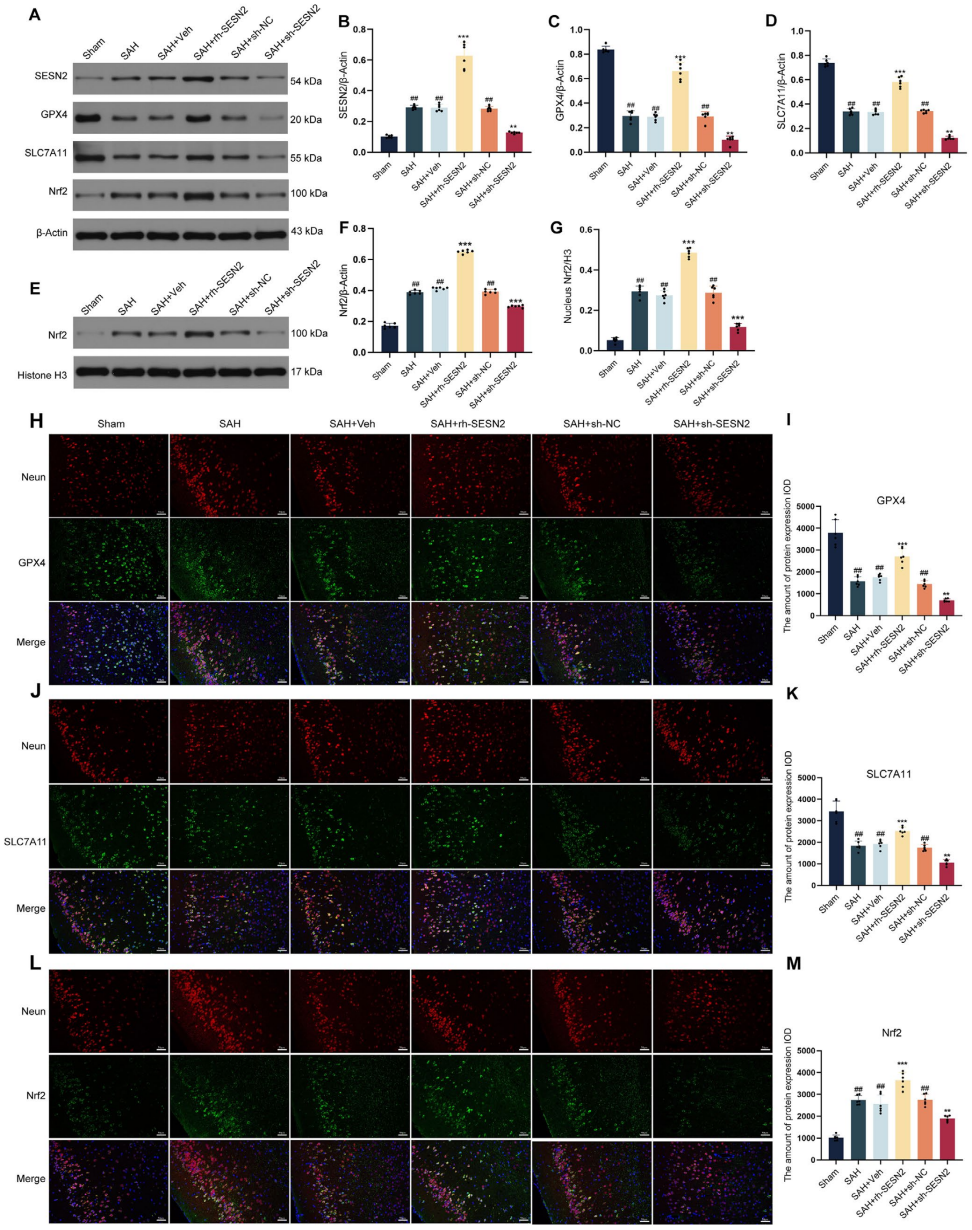
SESN2 activates the Nrf2 signaling pathway and inhibits ferroptosis in SAH mice. (A) Western blot bands showing protein expression of SESN2, GPX4, SLC7A11, Nrf2, and β‐Actin across different experimental groups. (B–D) Quantitative analysis of SESN2 (B), GPX4 (C), and SLC7A11 (D) expression normalized to β‐Actin. (E) Western blot bands showing nuclear Nrf2 expression and Histone H3 loading control. (F, G) Quantitative analysis of total Nrf2 normalized to β‐Actin (F) and nuclear Nrf2 normalized to Histone H3 (G). (H‐M) Immunofluorescence analysis: (H) Co‐localization of NeuN (red) and GPX4 (green) with DAPI (blue) nuclear staining; (I) Quantification of GPX4 fluorescence intensity. (J) Co‐localization of NeuN (red) and SLC7A11 (green) with DAPI (blue); (K) Quantification of SLC7A11 fluorescence intensity. (L) Co‐localization of NeuN (red) and Nrf2 (green) with DAPI (blue); (M) Quantification of Nrf2 fluorescence intensity. Data presented as mean ± SD. *n* = 6 per group. #*p* < 0.05, ##*p* < 0.01 vs. Sham group; *p < 0.05, **p < 0.01, ***p < 0.001 vs. SAH group. Scale bar = 50 μm.

To investigate the molecular mechanisms underlying SESN2‐mediated neuroprotection against ferroptosis, we examined key ferroptosis regulators following SAH induction. Western blot analysis (Figure 2A–DF) revealed that SAH induced increased endogenous SESN2 and Nrf2 expression but significantly decreased GPX4 and SLC7A11 levels, indicating both the activation of endogenous protective mechanisms and the occurrence of ferroptotic processes following SAH. Administration of exogenous rh‐SESN2 significantly increased the expression of GPX4, SLC7A11, and Nrf2, whereas SESN2 knockdown further reduced the expression of these proteins. Western blot analysis of nuclear Nrf2 protein levels (Figure 2E,G) demonstrated that SESN2 promoted enhanced nuclear translocation of Nrf2, suggesting increased transcriptional activity. Immunofluorescence staining (Figure 2H–K) revealed that SAH substantially diminished expression of GPX4 and SLC7A11 in NeuN‐positive cortical neurons relative to sham controls. Compared to SAH, exogenous rh‐SESN2 administration significantly increased the expression of these ferroptosis‐related proteins in neurons. Moreover, SAH significantly increased Nrf2 fluorescence intensity in cortical neurons relative to sham controls, and rh‐SESN2 administration further enhanced this effect. However, SESN2 knockdown markedly reduced Nrf2 expression compared with that in the SAH group (Figure 2L,M). These findings indicate that SESN2 alleviates oxidative stress and ferroptosis following SAH by enhancing Nrf2 nuclear translocation and transcriptional activity, leading to upregulation of antioxidant and antiferroptotic proteins GPX4 and SLC7A11.

### 3.3 SESN2 Attenuates Hemin‐Induced Oxidative Stress and Ferroptosis in HT22 Cells via Nrf2 Pathway Activation

**FIGURE 3:**
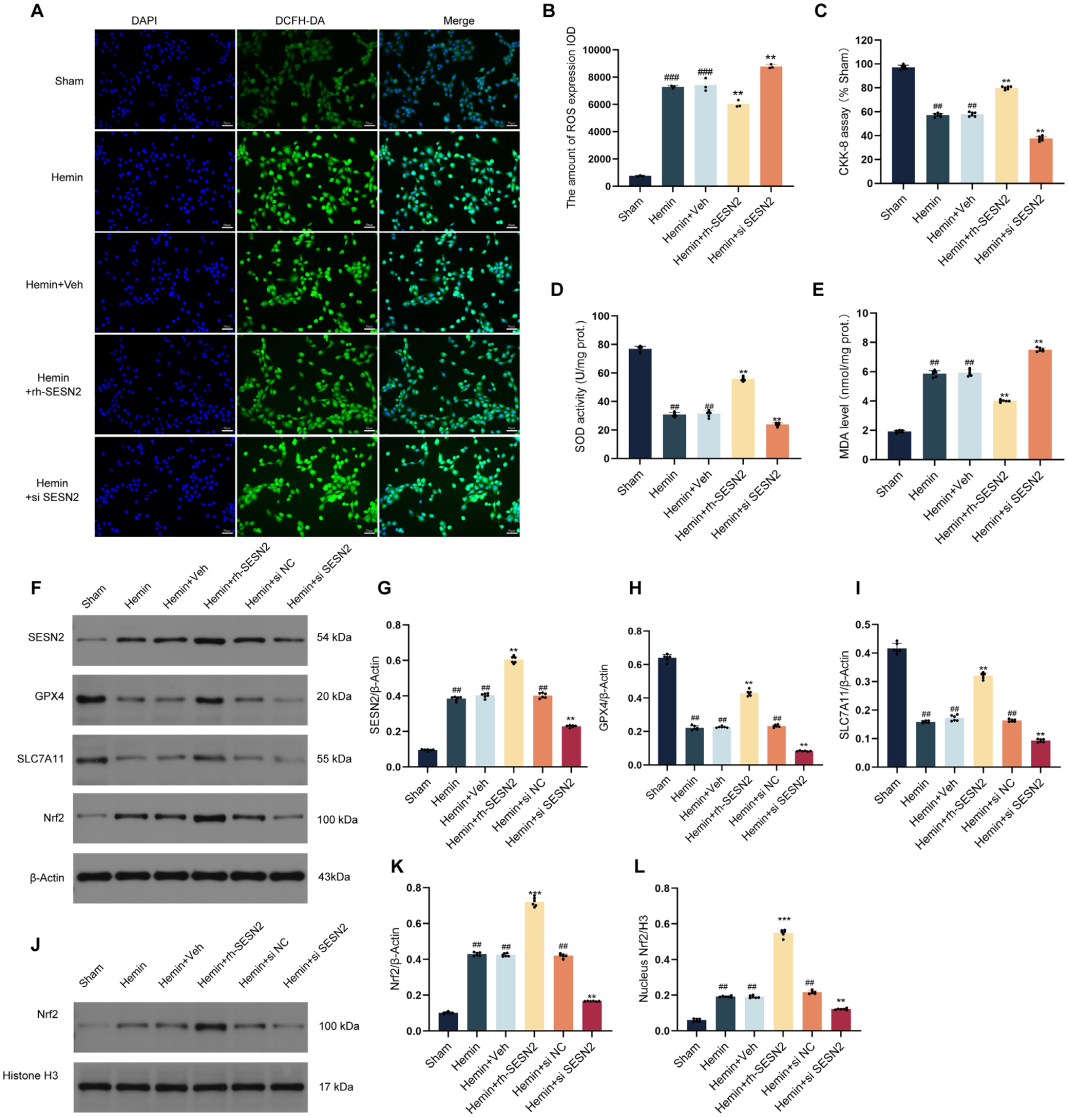
Effects of SESN2 on neuronal survival and oxidative stress markers in an in vitro SAH model. (A) Immunofluorescence images showing ROS production detected by DCFH‐DA staining (green) with DAPI nuclear staining (blue) and merged views across experimental groups. Scale bars = 20 μm. (B) Corresponding quantification of ROS levels in each experimental group, *n* = 3 per group. (C) Cell viability of HT22 cells assessed by CCK‐8 assay, *n* = 6 per group. (D, E) Quantitative analyses of SOD activity and MDA content, *n* = 6 per group. (F) Western blot analysis and quantitative analysis of SESN2 (G), GPX4 (H), SLC7A11 (I) and Nrf2 (K), *n* = 6 per group. (J, L) Western blot analysis and quantification of nuclear Nrf2, *n* = 6 per group. Data indicated as mean ± SD. ##*p* < 0.01, ###*p* < 0.001 vs. Sham group, **p < 0.01 vs. Hemin group.

**FIGURE 4:**
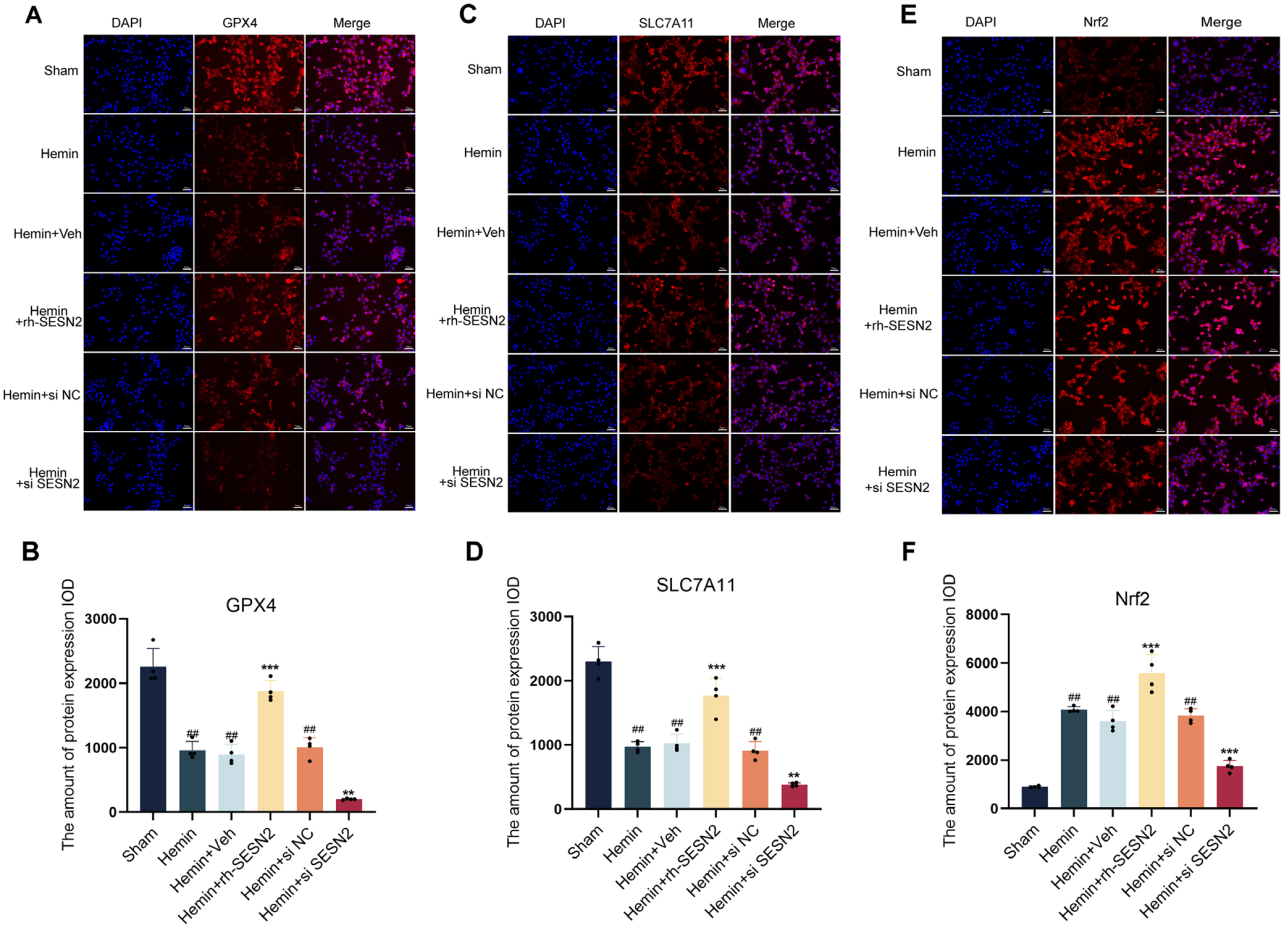
Immunofluorescence analysis of GPX4 and SLC7A11 expression in HT22 cells after SAH in vitro. (A, B) Immunofluorescence staining of GPX4 in HT22 neurons. DAPI (blue), GPX4 (red), and merged images shown across different groups with corresponding expression quantification. (C, D) Immunofluorescence staining of SLC7A11 in HT22 neurons. DAPI (blue), SLC7A11 (red), and merged images shown with quantification. (E, F) Immunofluorescence staining of Nrf2 in HT22 neurons. Data indicated as mean ± SD. *n* = 4 per group. ##*p* < 0.01 vs. Sham group, **p < 0.01 vs. Hemin group. Scale bar = 50 μm.

To further validate our in vivo findings, we established an in vitro SAH model using hemin‐treated HT22 cells. DCFH‐DA staining (Figure 3A) demonstrated that hemin exposure significantly increased intracellular ROS production, as evidenced by increased fluorescence intensity. Compared with hemin treatment, rh‐SESN2 treatment substantially reduced the DCFH‐DA fluorescence intensity. Quantitative assessments (Figure 3B) further confirmed that hemin exposure induced a marked increase in ROS levels, while rh‐SESN2 treatment effectively attenuated hemin‐induced oxidative stress, restoring ROS levels close to those in the sham group. Moreover, quantitative analyses revealed that hemin exposure significantly reduced cell viability (Figure 3C). Importantly, these hemin‐induced changes were substantially ameliorated by rh‐SESN2 protein treatment but exacerbated by SESN2 knockdown. Similarly, hemin treatment decreased SOD activity (Figure 3D) but increased MDA levels (Figure 3E), which is indicative of oxidative stress, and these changes were significantly reversed by rh‐SESN2 treatment. Western blot analyses (Figure 3F–IK) confirmed that hemin treatment upregulated endogenous SESN2 and Nrf2 expression while reducing GPX4 and SLC7A11 levels. Importantly, rh‐SESN2 intervention significantly increased the expression of SESN2, Nrf2, GPX4, and SLC7A11, whereas SESN2 knockdown further suppressed these proteins. Western blot analyses (Figure 3F–L) confirmed that rh‐SESN2 intervention significantly increased the expression of SESN2, Nrf2, GPX4, and SLC7A11, while enhancing Nrf2 nuclear translocation. Immunofluorescence staining (Figure 4) further validated these findings, showing that rh‐SESN2 significantly increased the expression of Nrf2, GPX4, and SLC7A11 in hemin‐treated neurons. These cellular results are consistent with our in vivo observations, confirming that SESN2 attenuates hemin‐induced oxidative stress and ferroptosis by promoting Nrf2 nuclear translocation and transcriptional activity, thereby increasing the levels of the antioxidant and antiferroptotic proteins GPX4 and SLC7A11.

To further evaluate whether the observed cellular damage reflects ferroptosis, we performed complementary experiments probing ACSL4, a pro‐ferroptotic enzyme, and included Ferrostatin‐1 (Fer‐1) as a positive ferroptosis inhibitor control. HT22 cells were assigned to four groups: Control, Hemin, Hemin + rh‐SESN2, and Hemin + Fer‐1. Western blot analysis revealed that Hemin markedly increased ACSL4 protein levels relative to Control (*p* < 0.05). Treatment with rh‐SESN2 significantly attenuated the Hemin‐induced ACSL4 upregulation (*p* < 0.05). The Hemin + Fer‐1 group (positive control) showed a similar suppression of ACSL4 as observed with rh‐SESN2 (*p* < 0.05 vs. Hemin) (Figure S3). These data are consistent with our prior findings on GPX4 and SLC7A11 and with functional oxidative stress readouts, and they provide additional molecular evidence that Hemin triggers ferroptosis‐related changes that can be mitigated by rh‐SESN2 or a canonical ferroptosis inhibitor.

### 3.4 Nrf2 Inhibition by ML385 Markedly Attenuates SESN2‐Mediated Protection Against Ferroptosis in SAH Mice

**FIGURE 5:**
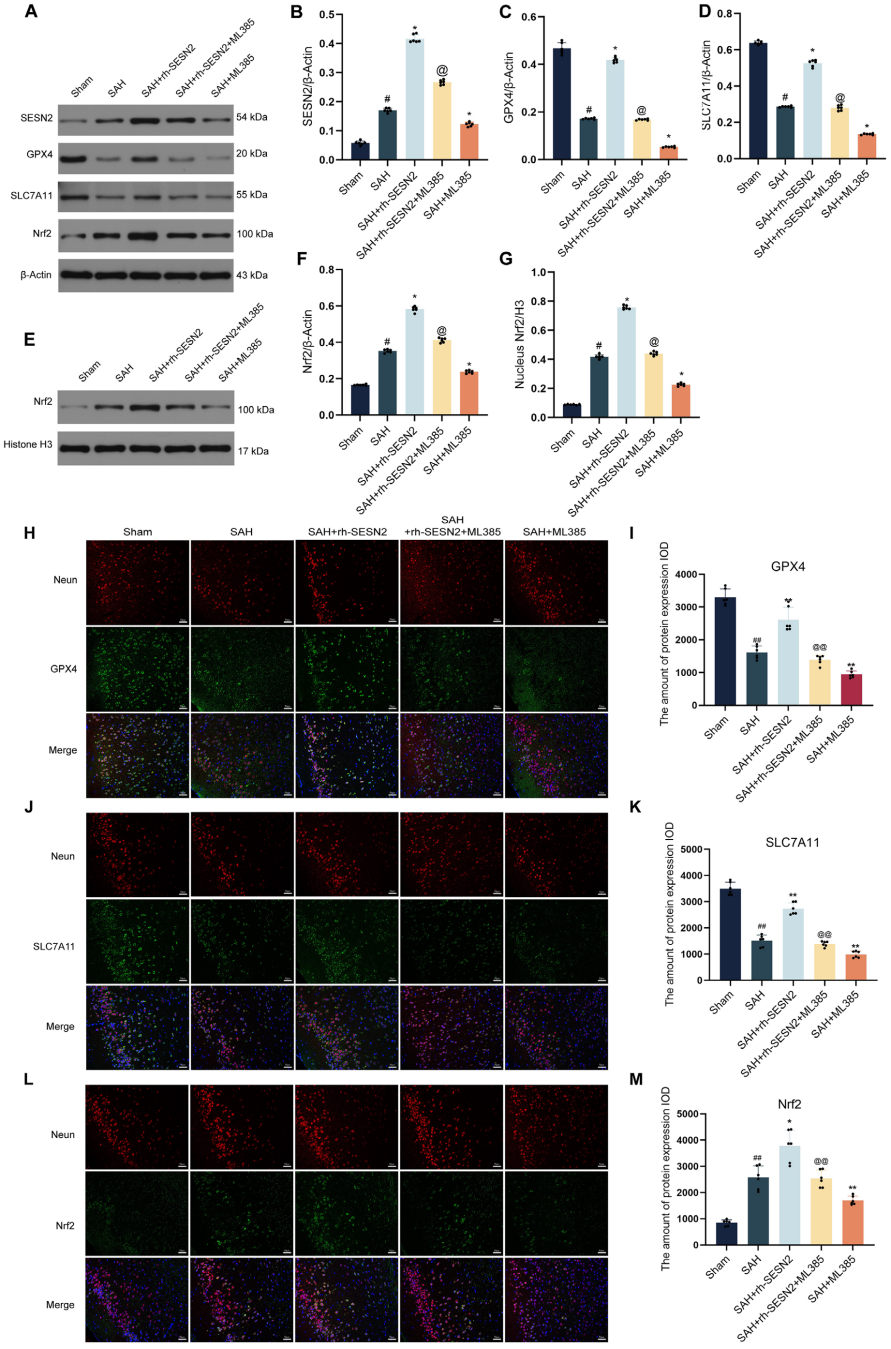
Nrf2 inhibition by ML385 markedly attenuates SESN2‐mediated protection against ferroptosis in SAH mice. (A) Western blot bands and quantitative analysis of SESN2 (B), GPX4 (C), SLC7A11 (D), and Nrf2 (F) normalized to β‐Actin across different experimental groups. (E, G) Western blot bands and quantitative analysis of nuclear Nrf2 expression normalized to Histone H3. (H‐M) Immunofluorescence analysis: (H) Co‐localization of NeuN (red) and GPX4 (green) with DAPI (blue) nuclear staining; (I) Quantification of GPX4 fluorescence intensity. (J) Co‐localization of NeuN (red) and SLC7A11 (green) with DAPI (blue); (K) Quantification of SLC7A11 fluorescence intensity. (L) Co‐localization of NeuN (red) and Nrf2 (green) with DAPI (blue); (M) Quantification of Nrf2 fluorescence intensity. Data indicated as mean ± SD. *n* = 6 per group. #*p* < 0.05, ##*p* < 0.01 vs. Sham group, *p < 0.05, **p < 0.01 vs. SAH group, @*p* < 0.05 vs. SAH + rh‐SESN2 group. Scale bar = 50 μm.

To determine whether Nrf2 activation is essential for the protective effects of SESN2, we administered ML385, a specific Nrf2 inhibitor. Western blot analysis confirmed that ML385 significantly attenuated the rh‐SESN2‐induced upregulation of GPX4, SLC7A11, total Nrf2, and inhibited Nrf2 nuclear translocation (Figure 5A–G). Immunofluorescence staining revealed that compared to the SAH + rh‐SESN2 group, the co‐localization of GPX4, SLC7A11, and Nrf2 with NeuN‐positive cortical neurons was significantly reduced in the SAH + rh‐SESN2 + ML385 group (Figure 5H–M), indicating that ML385 significantly attenuated the rh‐SESN2‐induced upregulation of GPX4, SLC7A11, and nuclear translocation of Nrf2. These findings suggest that Nrf2 activation and nuclear translocation function as critical downstream events in the SESN2‐mediated anti‐ferroptotic pathway.

### 3.5 In Vitro Confirmation That Nrf2 Inhibition Attenuates SESN2‐Mediated Protection Against Ferroptosis

**FIGURE 6:**
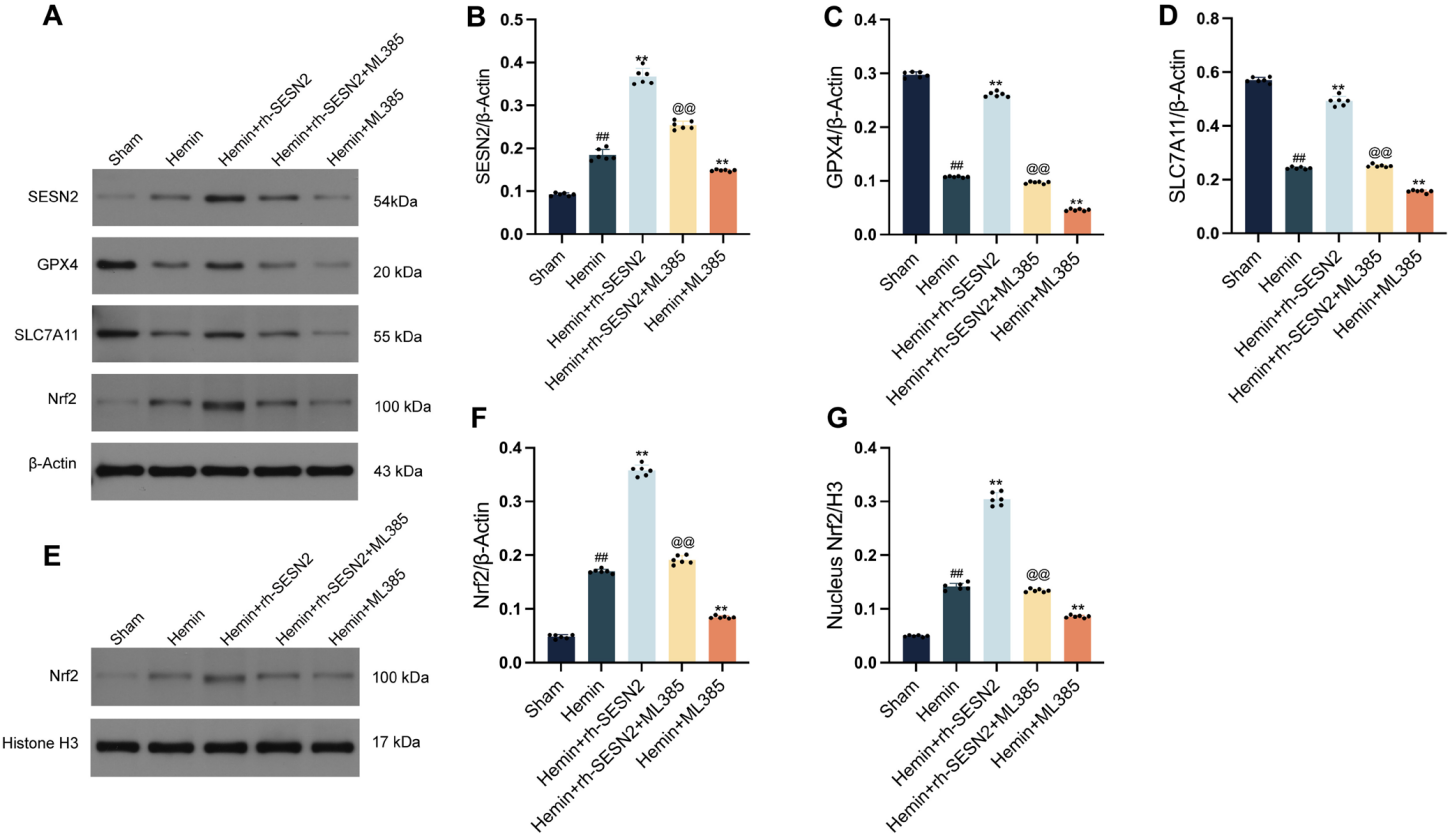
Nrf2 inhibition by ML385 abolishes SESN2‐mediated protection against ferroptosis in HT22 cells. (A) Western blot bands and quantitative analysis of SESN2 (B), GPX4 (C), SLC7A11 (D) and Nrf2 (F) normalized to β‐Actin across different experimental groups. (E, H) Western blot bands and quantitative analysis of nuclear Nrf2 expression normalized to Histone H3. Data indicated as mean ± SD. *n* = 6 per group. ##*p* < 0.01 vs. sham group, **p < 0.01 vs. Hemin group, @@*p* < 0.01 vs. Hemin + rh‐SESN2 group.

We further investigated this mechanism using the established hemin‐treated HT22 cell model. As previously demonstrated, rh‐SESN2 administration significantly increased GPX4 and SLC7A11 levels in hemin‐treated cells. However, cotreatment with ML385, which specifically binds to Nrf2 and prevents its nuclear translocation, significantly attenuated these protective effects, resulting in markedly lower expression of these key ferroptosis regulators than those in the rh‐SESN2 group (Figure 6A–D). This finding reinforces the idea that Nrf2 activation is crucial for mediating the protective effects of SESN2. Additionally, ML385 cotreatment with rh‐SESN2 significantly reduced both the total and inhibited Nrf2 nuclear translocation, which were elevated by rh‐SESN2 administration alone (Figure 6E–G). These pharmacological inhibition experiments provide direct evidence that Nrf2 nuclear translocation and subsequent transcriptional activation serve as the critical downstream mediator in the SESN2 protective pathway against ferroptosis under hemorrhagic conditions.

To complement the pharmacological inhibition data and to address potential off‐target concerns with ML385, we performed short‐term genetic knockdown of Nrf2 in HT22 cells using a validated siRNA (si‐Nrf2). Knockdown efficiency was confirmed by qPCR and Western blot (Figure S4A–C). Functionally, si‐Nrf2 markedly abrogated the ability of rh‐SESN2 to restore the antiferroptotic proteins GPX4 and SLC7A11 in hemin‐treated cells; these effects closely paralleled those observed with ML385 treatment (Figure S4D–I). Together, the pharmacologic and genetic inhibition data indicate that Nrf2 is required for the rh‐SESN2 mediated upregulation of key antiferroptotic proteins in this model.

### 3.6 SESN2 Protects Against Ferroptosis Through the AMPKα/PGC1α/Nrf2 Signaling Axis in Hemin‐Treated HT22 Cells In Vitro

**FIGURE 7:**
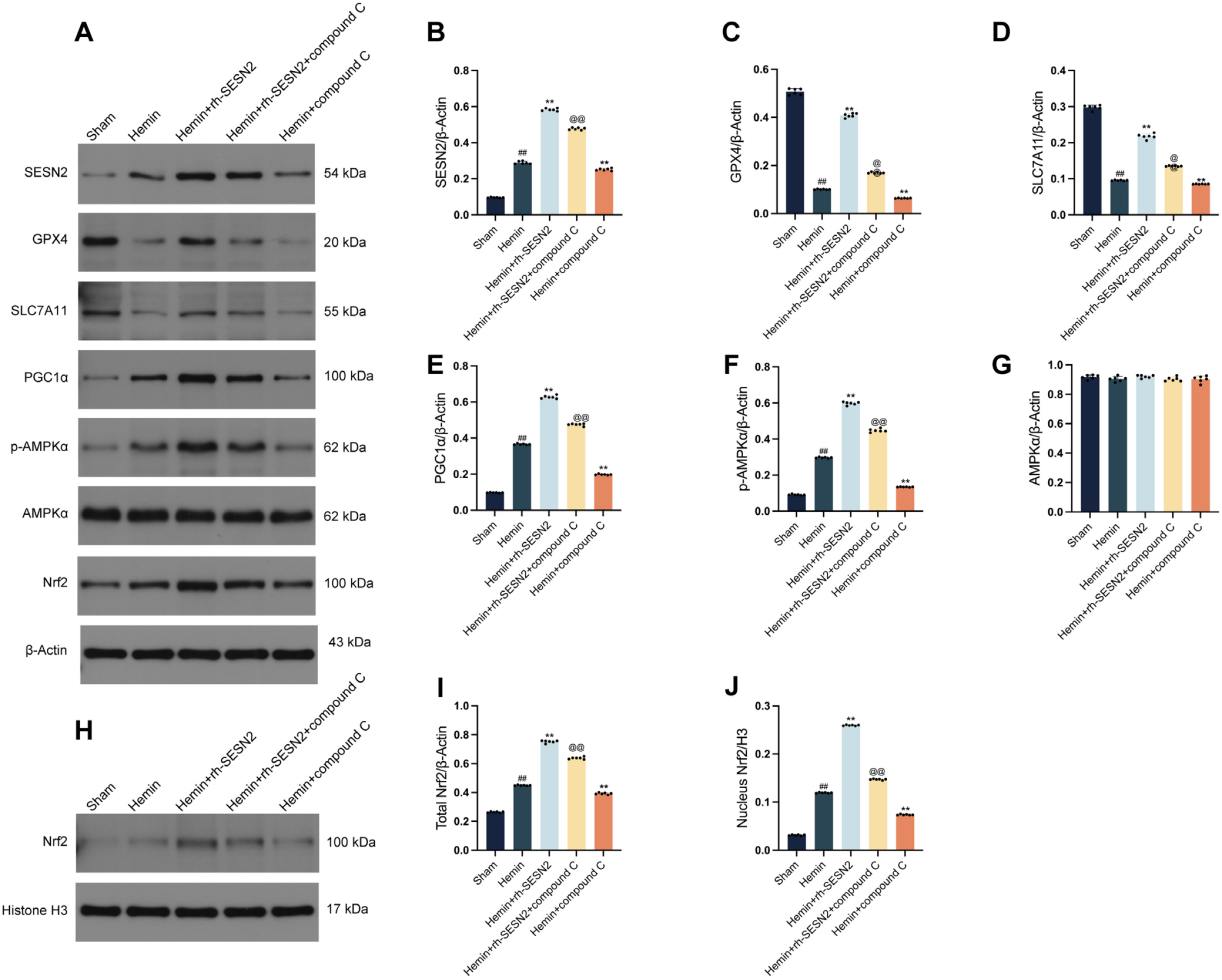
AMPKα inhibition by Compound C partially reverses SESN2‐mediated protection against ferroptosis in HT22 cells. (A) Western blot bands and quantitative analysis of SESN2 (B), GPX4 (C), SLC7A11 (D), PGC1α (E), p‐AMPKα (F), AMPKα (G), and Nrf2 (I) normalized to β‐Actin across different experimental groups. (H, J) Western blot bands and quantitative analysis of nuclear Nrf2 expression normalized to Histone H3. Data indicated as mean ± SD. *n* = 6 per group. ##*p* < 0.01 vs. sham group, **p < 0.01 vs. Hemin group, @@*p* < 0.01 vs. Hemin + rh‐SESN2 group.

After establishing the critical role of Nrf2 in mediating the protective effects of SESN2, we next investigated upstream regulators of this pathway. Western blot analysis (Figure 7) revealed that, compared with sham treatment, hemin exposure significantly increased phosphorylated AMPKα (p‐AMPKα) levels, PGC1α expression, and Nrf2 nuclear translocation in HT22 cells, likely indicating an endogenous protective response to oxidative stress. Moreover, hemin treatment suppressed the expression of the antiferroptotic proteins GPX4 and SLC7A11. Treatment with rh‐SESN2 further significantly increased the activation of p‐AMPKα, PGC1α, and enhanced Nrf2 nuclear translocation but also substantially restored GPX4 and SLC7A11 expression, which were suppressed by hemin. To confirm the role of AMPKα in this protective pathway, we pretreated cells with Compound C, a specific AMPKα inhibitor, followed by rh‐SESN2 treatment prior to hemin administration. Notably, Compound C significantly attenuated the rh‐SESN2‐mediated upregulation of GPX4 and SLC7A11 while simultaneously blocking AMPKα phosphorylation and partially suppressing the downstream activation of PGC1α and Nrf2 nuclear translocation. These findings establish a clear signaling cascade in which SESN2 activates AMPKα, which subsequently enhances PGC1α activity to facilitate Nrf2 nuclear translocation and transcriptional activation, ultimately upregulating antioxidant and antiferroptotic proteins to protect neurons from ferroptosis following SAH.

To test whether activation of downstream effectors can bypass AMPK inhibition, we conducted rescue experiments in HT22 cells with the following groups: Sham, Hemin, Hemin + rh‐SESN2, Hemin + rh‐SESN2 + Compound C, and Hemin + rh‐SESN2 + Compound C + sulforaphane (SFN, Nrf2 activator). AMPK was inhibited with Compound C and Nrf2 activated with SFN. As shown in Figure S5, Hemin decreased GPX4 and SLC7A11 protein levels, while rh‐SESN2 restored them. Compound C significantly attenuated the rh‐SESN2‐induced restoration of GPX4 and SLC7A11. Co‐treatment with SFN under AMPK inhibition partially recovered GPX4 and SLC7A11 expression compared with the Compound C group (*p* < 0.01), but did not fully return the levels to those seen with rh‐SESN2 without AMPK inhibition. These results suggest that pharmacologic activation of Nrf2 can at least partially compensate for AMPK blockade and restore antiferroptotic markers.

### 3.7 Inhibition of PGC1α by SR18292 Partially Reverses SESN2‐Mediated Protection Against Ferroptosis in HT22 Cells Exposed to Hemin In Vitro

**FIGURE 8:**
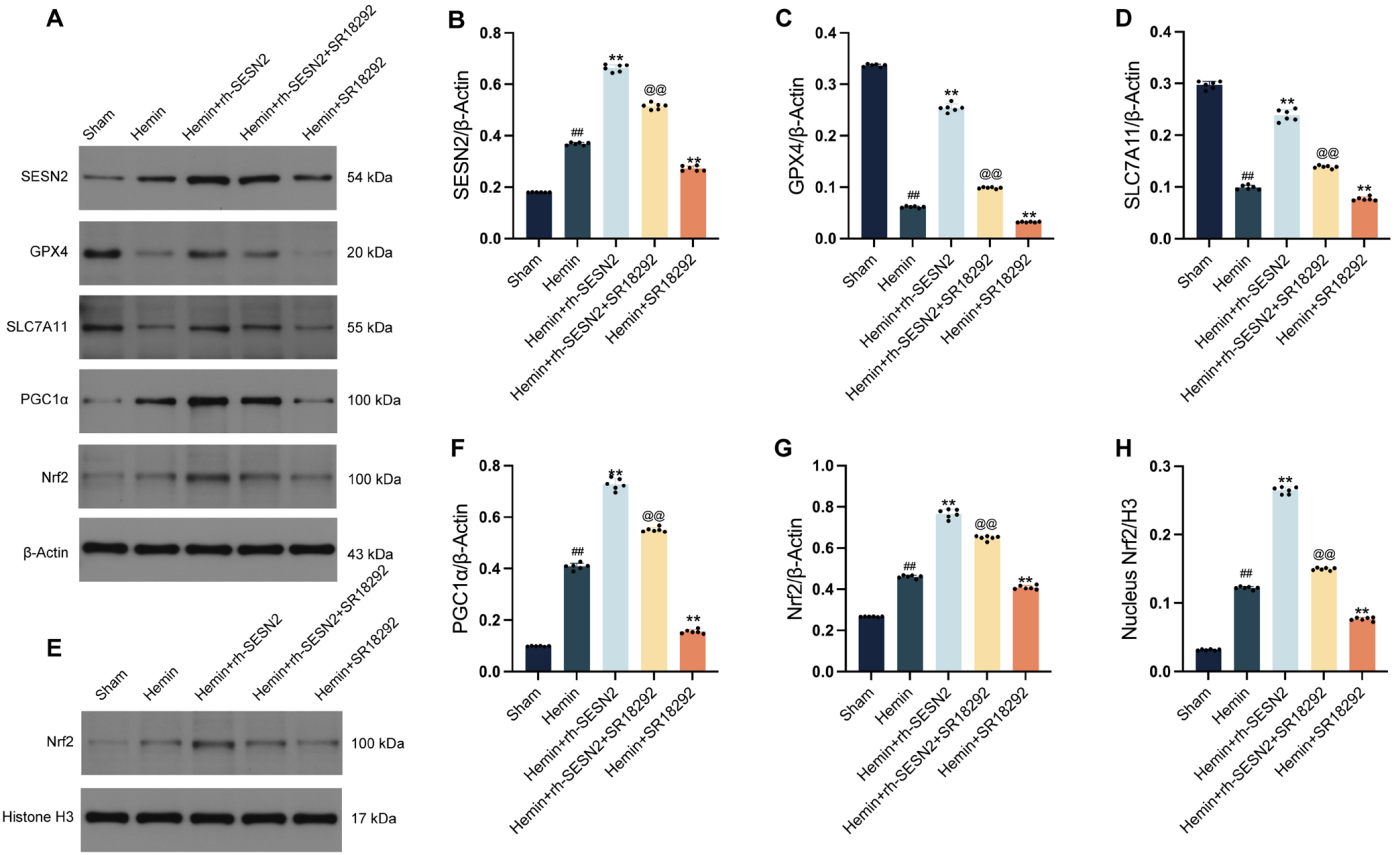
PGC1α inhibition by SR18292 partially reverses SESN2‐mediated protection against ferroptosis in HT22 cells. (A) Western blot bands and quantitative analysis of SESN2 (B), GPX4 (C), SLC7A11 (D), PGC1α (F), and Nrf2 (G) normalized to β‐Actin across different experimental groups. (E, H) Western blot bands and quantitative analysis of nuclear Nrf2 expression normalized to Histone H3. Data indicated as mean ± SD. *n* = 6 per group. ##*p* < 0.01 vs. sham group, **p < 0.01 vs. Hemin group, @@*p* < 0.01 vs. Hemin + rh‐SESN2 group.

To further elucidate PGC1α's specific role in the SESN2‐mediated protective pathway, we used SR18292, a specific PGC1α inhibitor. Western blot analysis (Figure 8) revealed that exogenous rh‐SESN2 significantly protected against hemin‐induced ferroptosis in HT22 cells by upregulating GPX4 and SLC7A11, which was consistent with our previous observations. Importantly, pretreatment with SR18292 prior to rh‐SESN2 administration and hemin exposure substantially diminished the protective effects of SESN2. SR18292 treatment markedly attenuated the SESN2‐induced Nrf2 nuclear translocation and the subsequent upregulation of the antiferroptotic proteins GPX4 and SLC7A11. These results establish PGC1α as an essential component in the SESN2‐initiated protective cascade against ferroptosis in neuronal cells. The significant impact of PGC1α inhibition on the protective function of SESN2 highlights the critical role of this transcriptional coactivator in neuronal protection against ferroptotic injury.

## 4 Discussion

In this study, we explored SESN2's contribution to EBI following SAH and investigated the underlying molecular mechanisms. Our findings demonstrate that (1) rh‐SESN2 administration mitigates neurological dysfunction, cerebral edema, blood–brain barrier compromise, and neuronal ferroptosis after SAH; (2) SESN2 exerts potent antioxidant effects and prevents iron accumulation; (3) SESN2 inhibits ferroptosis through activation of the AMPK/PGC1α/Nrf2 signaling cascade; and (4) inhibition of a component in this pathway partially reverses the neuroprotective effects of SESN2. These findings provide new insight into SAH pathophysiology and highlight SESN2 as a possible therapeutic target.

SESN2, a stress‐inducible protein with established cytoprotective functions, mitigates oxidative stress and regulates cellular metabolism [38]. Our research revealed a notable increase in SESN2 expression following SAH, consistent with findings across other acute CNS injuries. Song et al. reported increased SESN2 expression after traumatic brain injury, whereas Xie et al. reported similar upregulation in ischemic stroke models [47, 48]. These findings suggest that SESN2 upregulation represents an endogenous protective response to brain injury.

Our experiments demonstrated that exogenous rh‐SESN2 protein administration reduced neural deficits, cerebral edema, and BBB permeability at 24 h post SAH. The BBB, crucial for central nervous system stability, maintains tight junction components including ZO‐1 and occludin. These findings corroborate the findings of Shi et al., who reported similar BBB protective effects of SESN2 in hypoxic–ischemic brain injury [49]. Our electron microscopy observations revealed that SESN2 preserved mitochondrial morphology and integrity, essential for maintaining cellular energy homeostasis. These results establish a causal relationship between SESN2 deficiency and SAH‐induced neuronal damage.

Recent research has revealed ferroptosis, a form of programmed cell death dependent on iron and marked by oxidative damage to lipids, significantly contributes to neuronal injury after SAH [50, 51]. Ferroptosis has been implicated in several neurological conditions including cerebral ischemia, brain trauma, and hemorrhage [52, 53]. Our results revealed significant increases in iron deposition and peroxidized lipids, alongside decreased levels of ferroptosis inhibitors (GPX4 and SLC7A11) in both SAH mice and hemin‐treated neurons, confirming ferroptosis involvement in SAH pathophysiology. The interplay between ferroptosis and BBB disruption appears critical in SAH pathology [54]. In future studies, we will further explore SESN2's function in endothelial cells and other BBB components, as interactions within the neurovascular unit may significantly impact SAH pathology.

The anti‐ferroptotic effects of SESN2 are particularly noteworthy. Ferroptosis is characterized by GPX4‐mediated defense failure against lipid peroxidation and dysregulated iron metabolism [55]. Our results showed that SESN2 treatment markedly enhanced GPX4 and SLC7A11 expression, reduced lipid peroxidation, and prevented iron accumulation in SAH. Contemporary research has identified ferroptosis as a therapeutic target after SAH. Kang et al. reported that ferroptosis inhibitor ferrostatin‐1 reduced neuronal death following SAH [56], while Gao et al. demonstrated increased GPX4 expression protected against SAH‐induced EBI [57]. Our findings identify SESN2 as an endogenous regulator of ferroptosis in the context of SAH.

At the molecular level, we identified the AMPK/PGC1α/Nrf2 signaling pathway as mediating SESN2's neuroprotective effects. AMPK functions as a cellular energy sensor increasingly recognized for its neuroprotective capabilities across various neurological conditions [58]. PGC1α, activated downstream of AMPK, orchestrates mitochondrial biogenesis and antioxidant defense systems [58]. Nrf2, a master regulator of antioxidant responses, collaborates with PGC1α to amplify the expression of antioxidant and detoxifying enzymes [59]. Our data demonstrated that SESN2 treatment significantly increased AMPK phosphorylation, PGC1α expression, and Nrf2 nuclear translocation in SAH models. Importantly, ML385 treatment inhibited both Nrf2 activity and reduced SESN2 expression, suggesting a potential feedback loop between these factors.

These observations align with recent studies in other disease models. For example, Shin et al. reported that SESN2 safeguards mitochondrial function against glucose deprivation‐induced cytotoxicity by activating the AMPK/Nrf2 pathway [60]. Similarly, Gao et al. demonstrated that SESN2 alleviated ferroptosis in diabetic nephropathy through AMPK/PGC1α‐dependent mechanisms [61]. While our study highlights SESN2's role in regulating ferroptosis after SAH, it likely exerts additional neuroprotective effects through autophagy regulation, mTOR signaling, and mitochondrial homeostasis – aspects warranting further investigation [62].

It is important to recognize certain constraints of this investigation. First, our study focused primarily on EBI. However, SAH pathophysiology also includes delayed processes occurring over days to weeks that largely determine long‐term outcomes. Therefore, while rh‐SESN2 produced clear protection at 24 h, these results cannot be directly extrapolated to delayed ischemic events or long‐term functional recovery. Future work will extend the temporal window (e.g., 14, 21, and 28 days) to assess long‐term behavioral outcomes and chronic neuronal loss. Second, we exclusively used male mice in our experiments, which limits the generalizability of our findings across sexes. Given the known sex differences in stroke pathophysiology and treatment responses, future studies should include female animals to determine whether SESN2's protective effects are sex‐dependent. Third, our mechanistic findings derived mainly from in vitro models may not completely capture the intricate in vivo milieu that develops following SAH. The role of SESN2 in different neural cell types (neurons, astrocytes, microglia) requires further exploration, and cell type‐specific conditional knockout models could help delineate SESN2's function across different neural populations. Fourth, the precise mechanisms underlying SESN2‐mediated AMPK activation after SAH require further elucidation. Finally, our use of rh‐SESN2 protein rather than AAV‐mediated gene delivery may influence therapeutic efficacy due to potential limitations in intracellular transport. Future studies should compare these approaches to optimize SESN2‐based interventions.

In conclusion, our findings reveal that SESN2 activates the AMPK/PGC1α/Nrf2 axis to reduce oxidative stress and inhibit neuronal ferroptosis, thereby exerting neuroprotective effects against EBI following SAH. This work advances the understanding of SAH pathomechanisms and highlights SESN2 as a potential therapeutic candidate for addressing this severe neurological emergency. The elucidation of this protective molecular cascade offers new directions for developing targeted interventions against this devastating cerebrovascular condition.

## Author Contributions

Y.Y., X.C. conceived and designed the study. Y.Z., X.W., H.Z., Z.C. and R.Z. performed the experiments. S.X., W.W. and J.W. analyzed the data. Y.Z. and X.W. wrote the manuscript. Y.Y. supervised the study. Y.Z. and X.W. contributed equally to this research. All authors reviewed and approved the final version of the manuscript.

## Funding

This study was supported by National Natural Science Foundation of China (82002401), Natural Science Foundation of Jiangxi Province (20232BAB206066), Jiangxi Provincial Health Commission Science and Technology Plan Project (202410175), The Youth Talent Cultivation Project of First Affiliated Hospital of Nanchang University (YFYPY202281), Jiangxi Provincial Science and Technology Plan Project of Traditional Chinese Medicine (2023B0362), Jiangxi Provincial Graduate Innovation Fund Special Project (YC2024‐S057), Key Project of Postgraduate Research Topics of Jiangxi Science Education Society (2025KXJYS095).

## Ethics Statement

All animal procedures were reviewed and approved by the Ethics Committee of Nanchang University at Nanchang, China (CDYFY‐IACUC‐202309QR036).

## Consent

The authors have nothing to report.

## Conflicts of Interest

The authors declare no conflicts of interest.

## Supporting information


**Figure S1:** Validation of SESN2 knockdown efficiency by shRNA in mice.
**Figure S2:** Validation of SESN2 knockdown efficiency by different siRNA sequences in cells.
**Figure S3:** ACSL4 expression and Ferrostatin‐1 rescue in Hemin‐treated HT22 cells. (A) Representative Western blots of ACSL4 in sham, Hemin, Hemin + rh‐SESN2 and Hemin + Fer‐1 groups. (B) Densitometric quantification of ACSL4 normalized to β‐actin. Data indicated as mean ± SD; *n* = 6 per group. **p < 0.01 vs. sham group; @@p < 0.01 vs. Hemin group.
**Figure S4:** Genetic knockdown of Nrf2 blocks the protective effects of rh‐SESN2 on antiferroptotic proteins in hemin‐treated HT22 cells.
**Figure S5:** Rescue of rh‐SESN2 effects by Nrf2 activation under AMPK inhibition. (A) Western blot bands and quantitative analysis of GPX4 (B), SLC7A11 (C), and total Nrf2 (E) normalized to β‐Actin across different experimental groups. (D) Western blot bands and quantitative analysis of nuclear Nrf2 (F) expression normalized to Histone H3. Data are mean ± SD, *n* = 6 per group. ##*p* < 0.01 vs. sham group; **p < 0.01 vs. Hemin group; @@*p* < 0.01 vs. Hemin + rh‐SESN2 group; &&*p* < 0.01 vs. Hemin + rh‐SESN2 + Compound group.

**Table S1:** Sequences of AAV‐shRNAs targeting murine SESN2.
**Table S2:** Sequences of siRNAs targeting murine SESN2 and Nrf2.

## Data Availability

The datasets used or analyzed during the current study are available from the corresponding author on reasonable request.
