# Supplementary material for: Sestrin2 Mitigates Neuronal Ferroptosis Following Subarachnoid Hemorrhage via Orchestration of the AMPK/PGC1α/Nrf2 Signaling Axis
Source: CNS Neurosci Ther. 2026 Apr 30;32(5):e70908. doi: 10.1002/cns.70908 (PMC13131070; doi:10.1002/cns.70908)
Supplement: Supplementary file 1 — Figure S1: Validation of SESN2 knockdown efficiency by shRNA in mice. Figure S2: Validation of SESN2 knockdown efficiency by different siRNA sequences in cells. Figure S3: ACSL4 expression and Ferrostatin‐1 rescue in Hemin‐treated HT22 cells. (A) Representative Western blots of ACSL4 in sham, Hemin, Hemin + rh‐SESN2 and Hemin + Fer‐1 groups. (B) Densitometric quantification of ACSL4 normalized to β‐actin. Data indicated as mean ± SD; n = 6 per group. **p < 0.01 vs. sham group; @@p < 0.01 vs. Hemin group. Figure S4: Genetic knockdown of Nrf2 blocks the protective effects of rh‐SESN2 on antiferroptotic proteins in hemin‐treated HT22 cells. Figure S5: Rescue of rh‐SESN2 effects by Nrf2 activation under AMPK inhibition. (A) Western blot bands and quantitative analysis of GPX4 (B), SLC7A11 (C), and total Nrf2 (E) normalized to β‐Actin across different experimental groups. (D) Western blot bands and quantitative analysis of nuclear Nrf2 (F) expression normalized to Histone H3. Data are mean ± SD, n = 6 per group. ##p < 0.01 vs. sham group; **p < 0.01 vs. Hemin group; @@p < 0.01 vs. Hemin + rh‐SESN2 group; &&p < 0.01 vs. Hemin + rh‐SESN2 + Compound group. [file CNS-32-e70908-s001.docx]

# Supplementary Figures

| 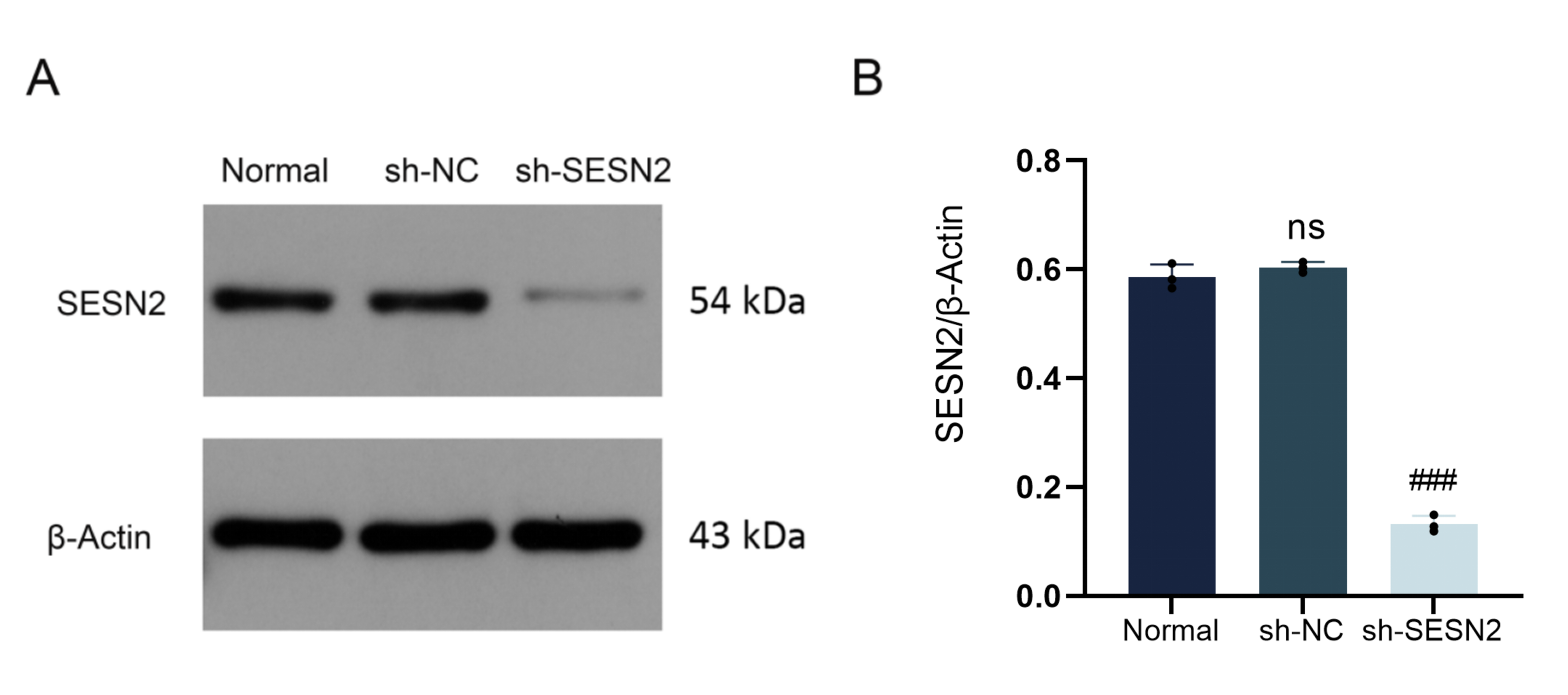 |
| --- |

**Figure S1. Validation of SESN2 Knockdown Efficiency by shRNA in mice.**

(A-B) **Western blot bands and quantitative analysis of SESN2 normalized to β-Actin across different experimental groups. Data indicated as mean ± SD. n=3 per group. ns p>0.05 vs. Normal group, ###p<0.01 vs. Normal group.**

| **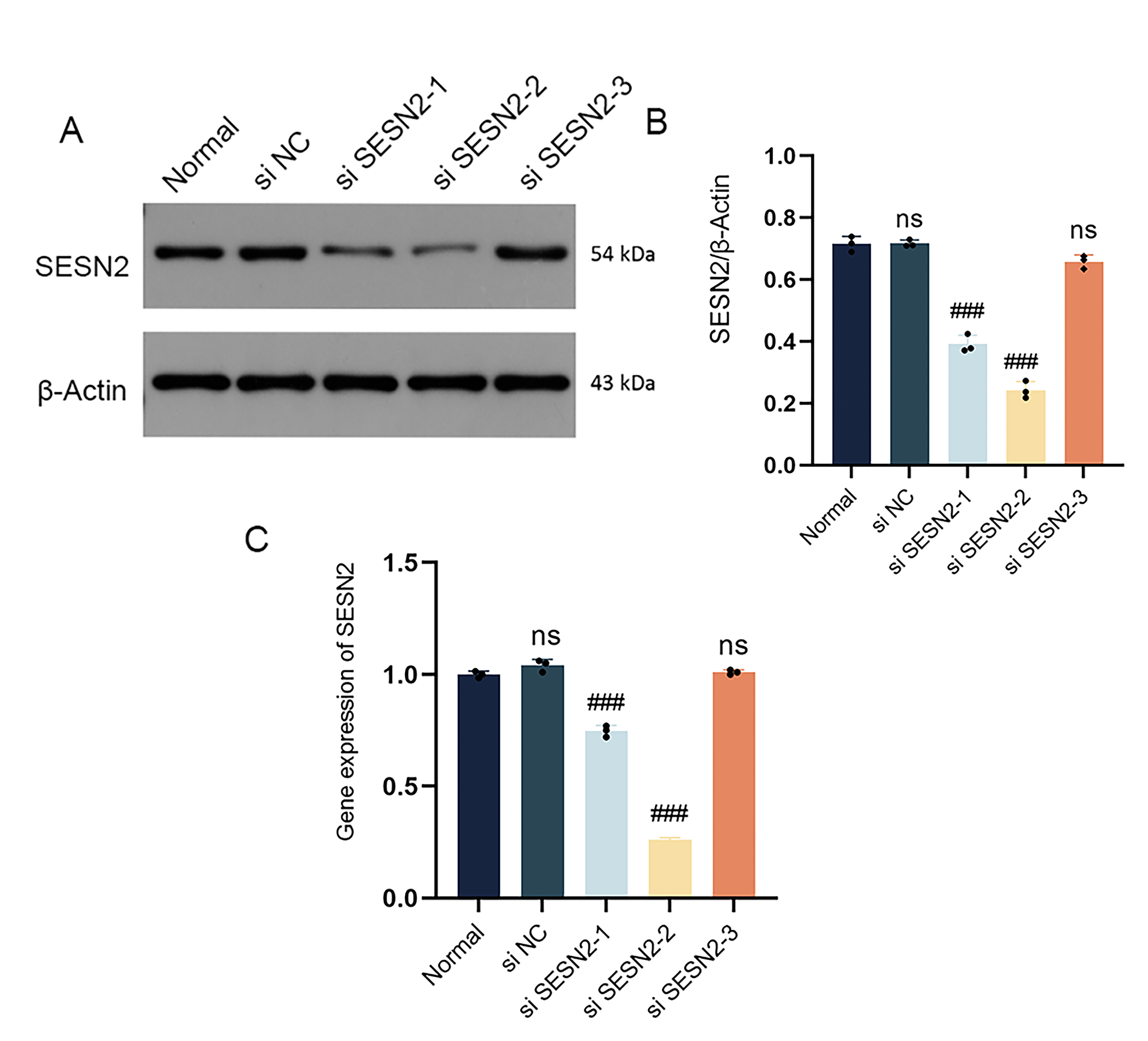** |
| --- |

**Figure S2. Validation of SESN2 Knockdown Efficiency by Different siRNA Sequences in Cells.**

1. B) **Western blot bands and quantitative analysis of SESN2 normalized to β-Actin across different experimental groups. (C) Quantitative analysis of SESN2 gene expression levels assessed by qRT-PCR. Data indicated as mean ± SD. n=3 per group. ns p>0.05 vs. Normal group, ###p<0.0101 vs. Normal group.**

| **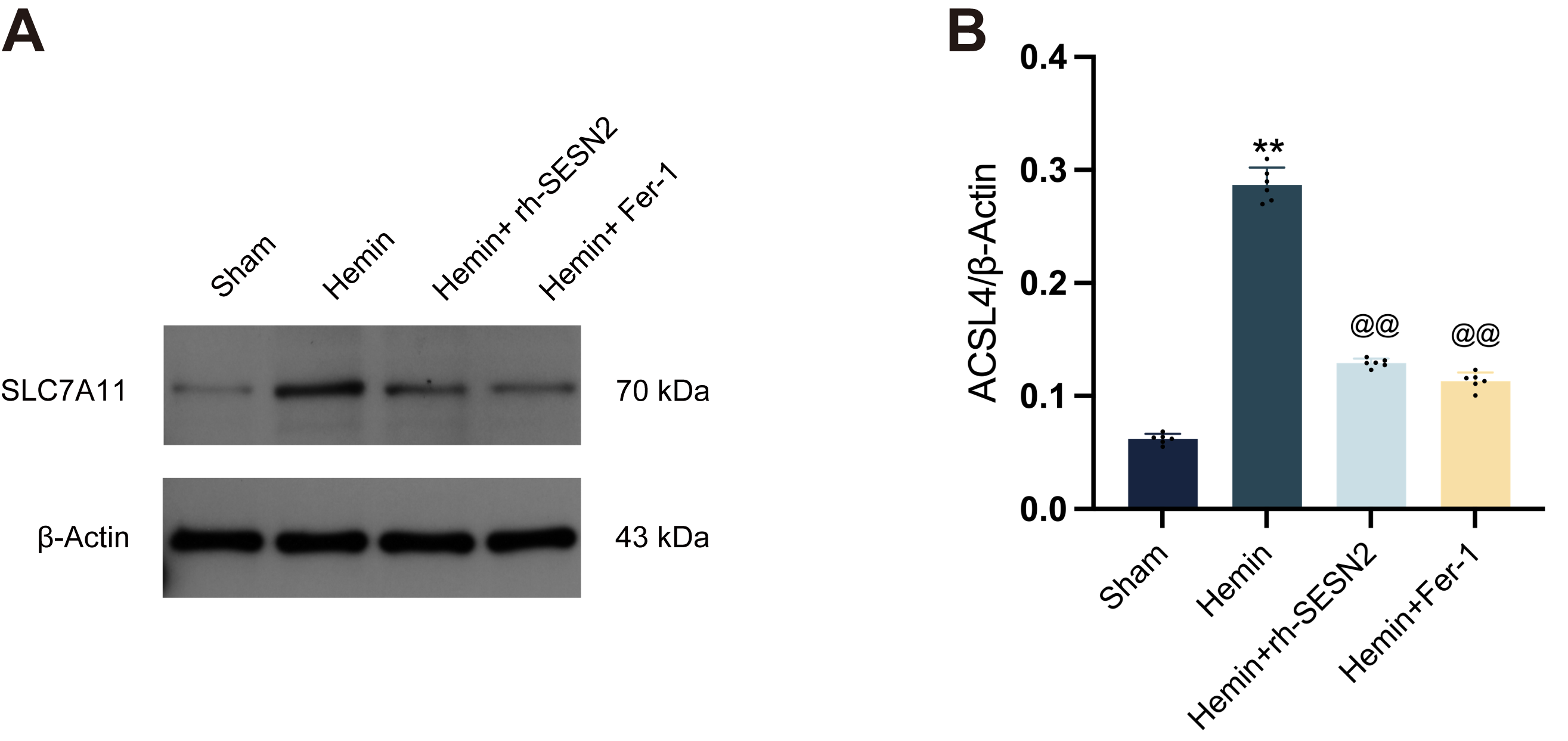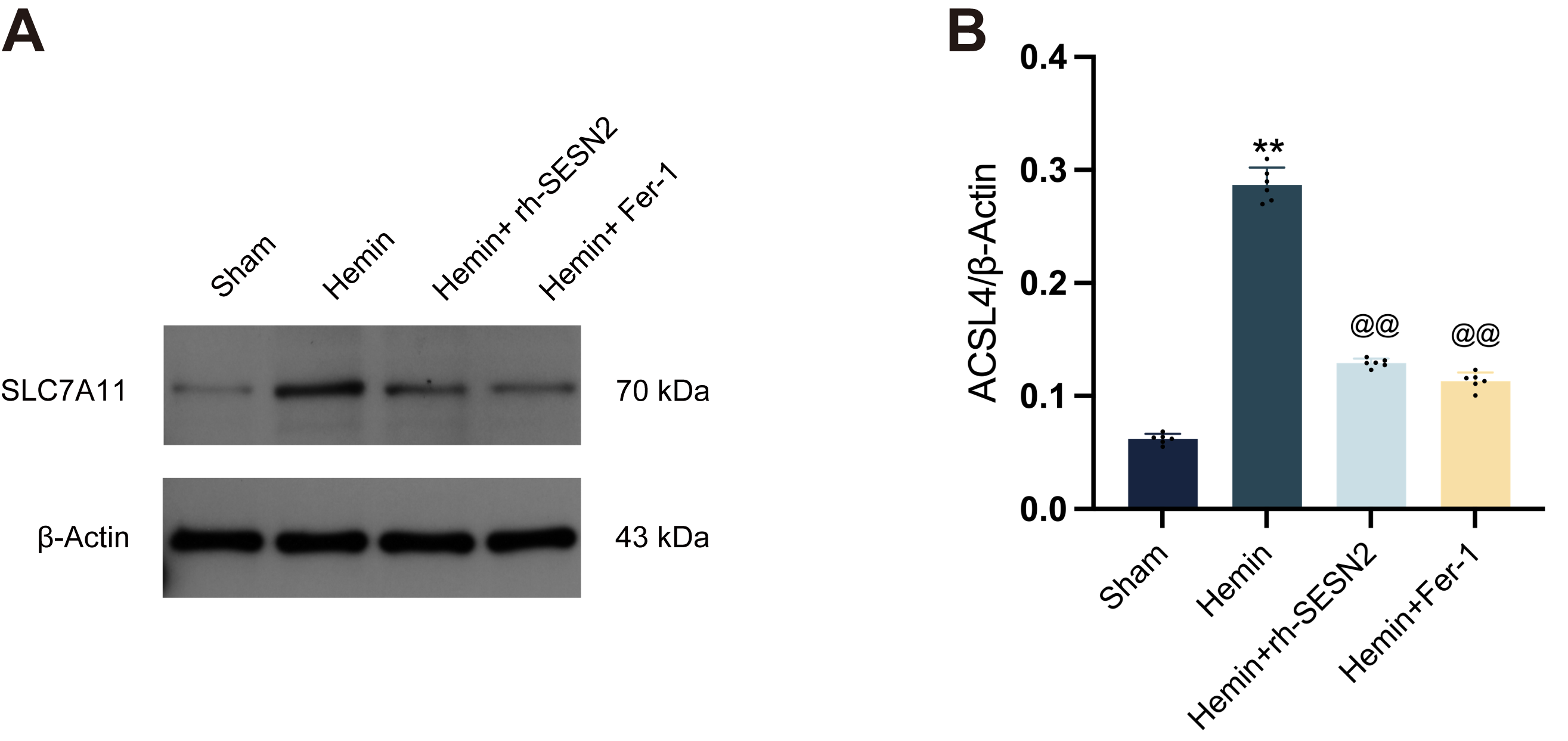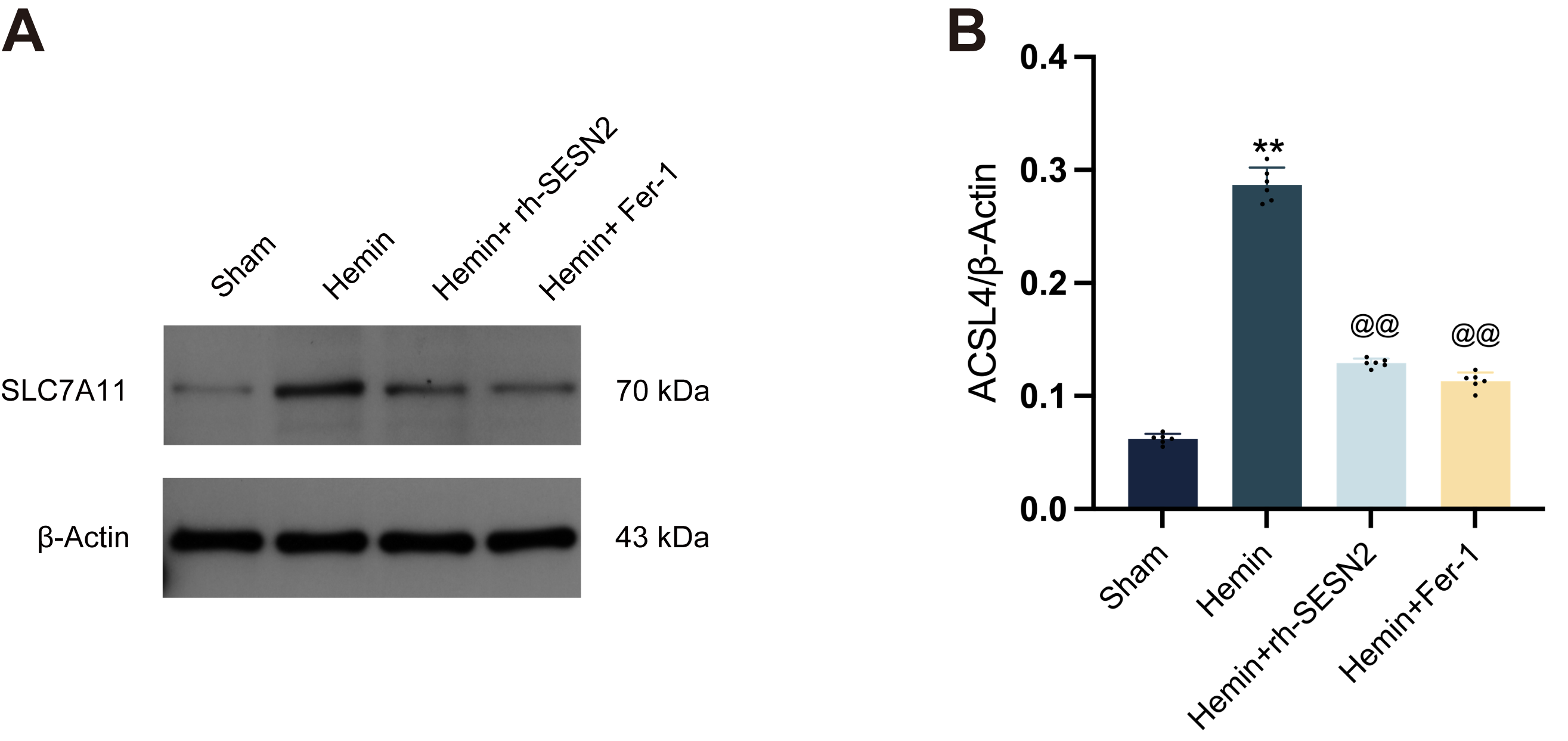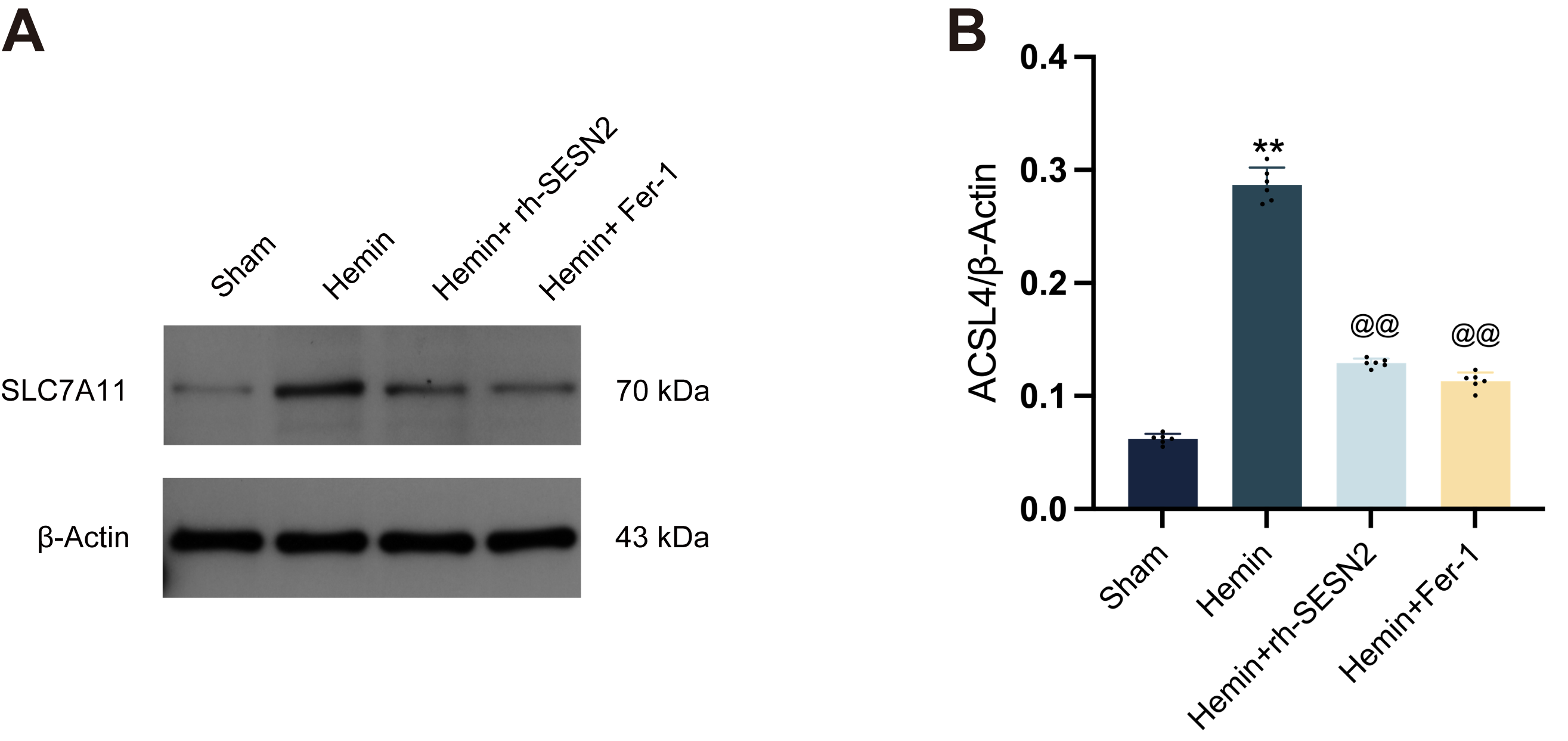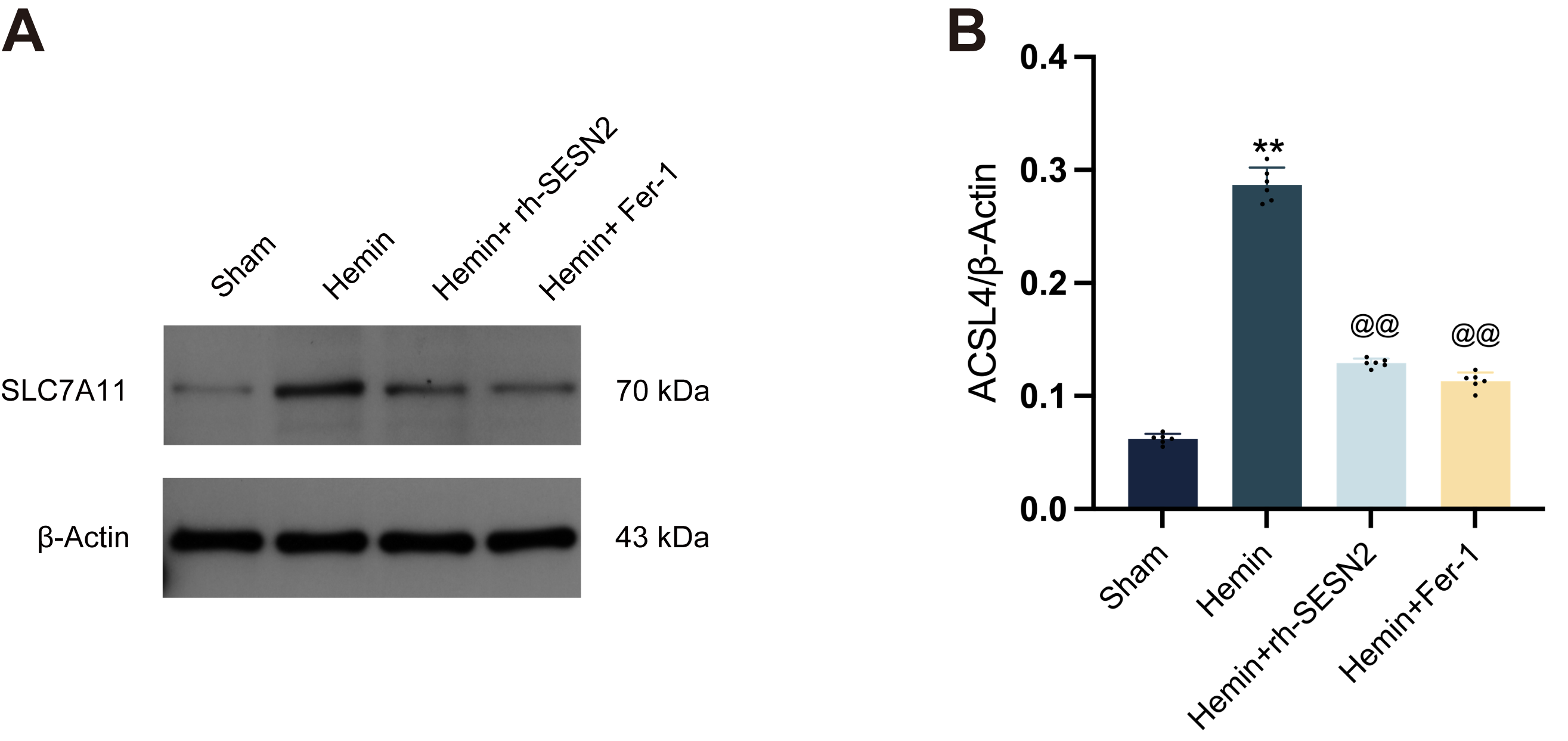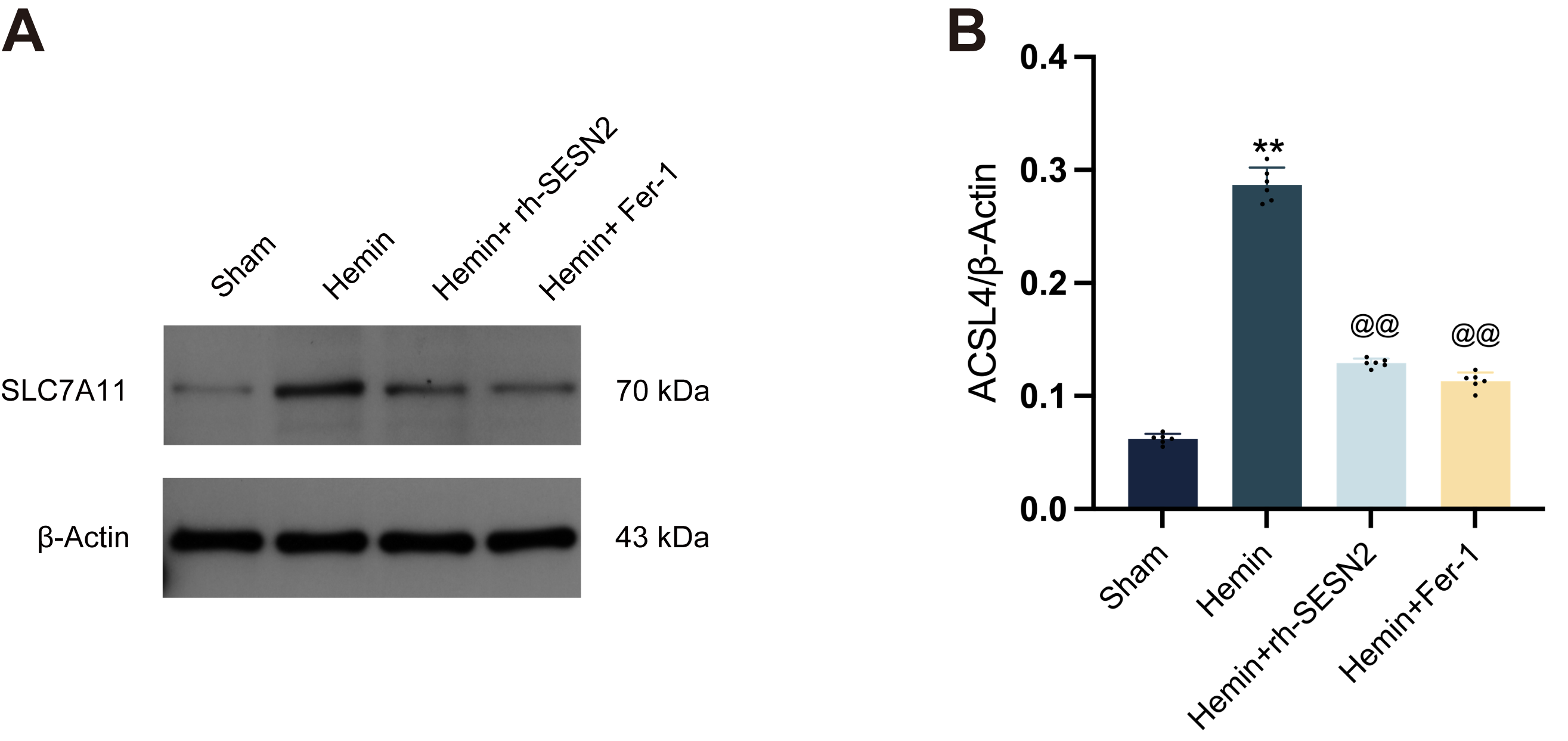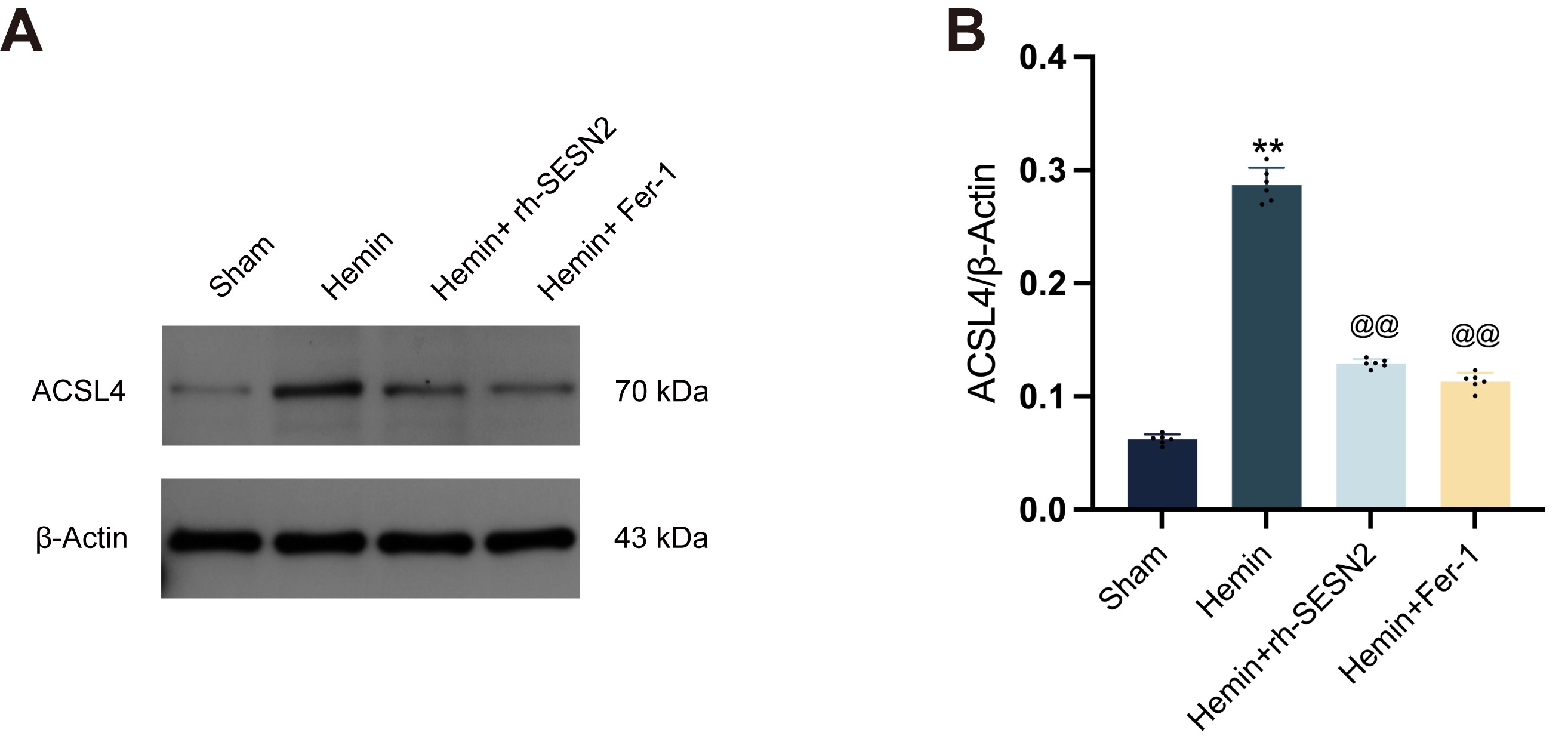**  ****Figure S3. ACSL4 expression and Ferrostatin‑1 rescue in Hemin‑treated HT22 cells.** (A) Representative Western blots of ACSL4 in sham, Hemin, Hemin + rh‑SESN2 and Hemin + Fer‑1 groups. (B) Densitometric quantification of ACSL4 normalized to β‑actin. Data indicated as mean ± SD; n = 6 per group. **p<0.01 vs. sham group; @@p<0.01 vs. Hemin group.** |
| --- |

| **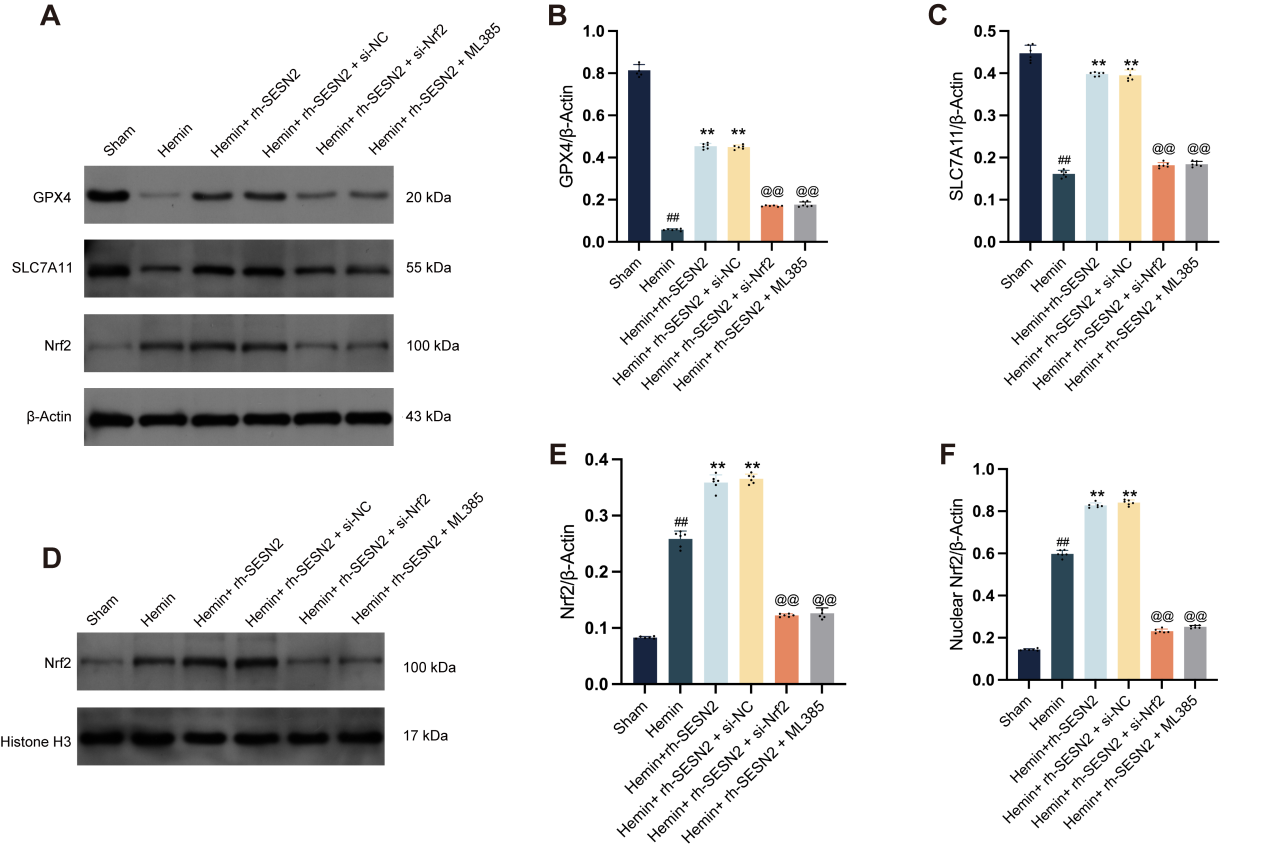**  ****Figure S4. Genetic knockdown of Nrf2 blocks the protective effects of rh‑SESN2 on antiferroptotic proteins in hemin‑treated HT22 cells.****  **(A) Western blot bands and quantitative analysis of GPX4 (B), SLC7A11 (C), and total Nrf2 (E) normalized to β-Actin across different experimental groups. (D) Western blot bands and quantitative analysis of nuclear Nrf2 (F) expression normalized to Histone H3. Data are mean ± SD, n = 6 per group. ##p<0.01 vs. sham group, **p<0.01 vs. Hemin group, @@p<0.01 vs. Hemin + rh-SESN2 group.** |
| --- |

| **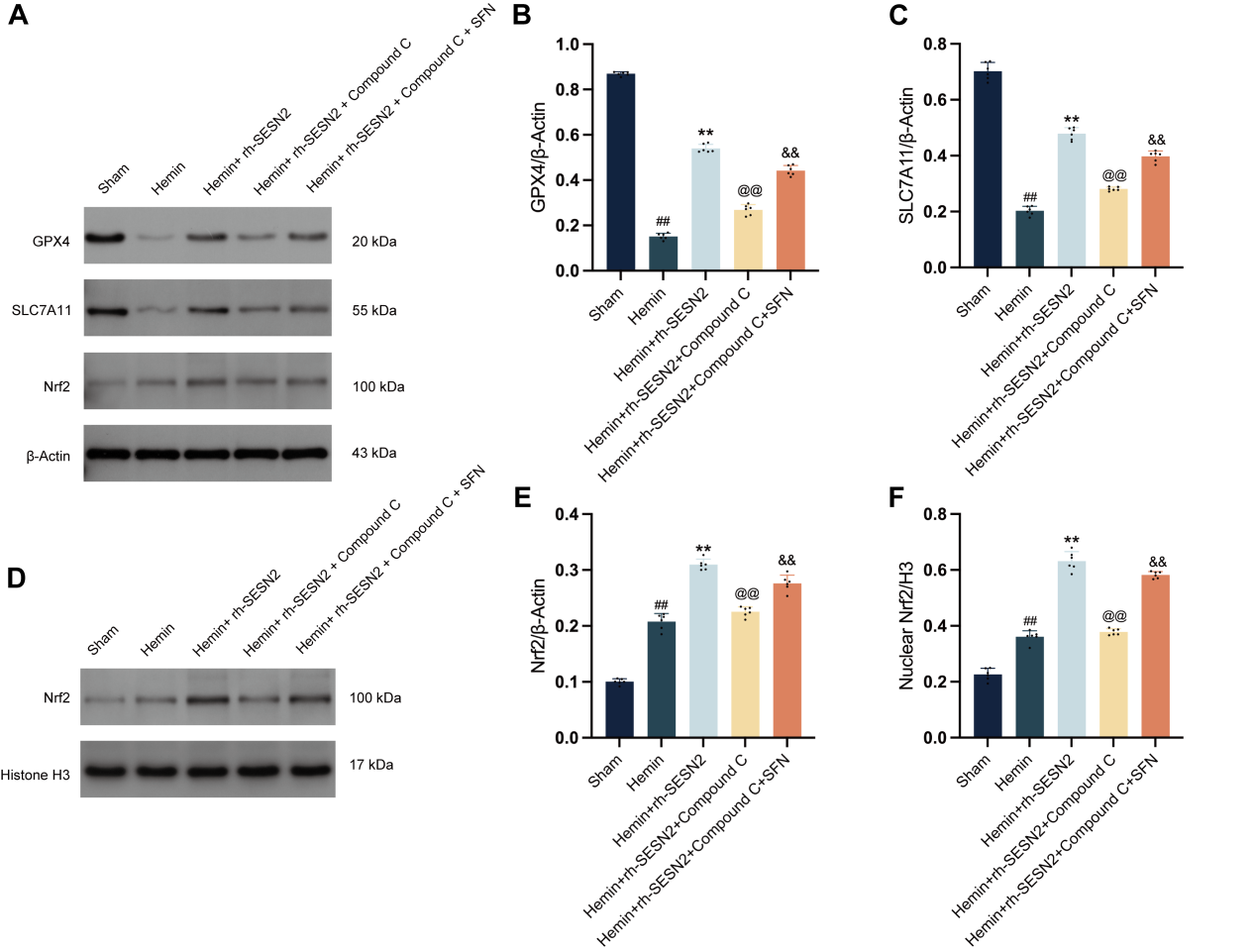**  ****Figure S5. Rescue of rh‑SESN2 effects by Nrf2 activation under AMPK inhibition.** (A) Western blot bands and quantitative analysis of GPX4 (B), SLC7A11 (C), and total Nrf2 (E) normalized to β-Actin across different experimental groups. (D) Western blot bands and quantitative analysis of nuclear Nrf2 (F) expression normalized to Histone H3. Data are mean ± SD, n = 6 per group. ##p<0.01 vs. sham group; **p<0.01 vs. Hemin group; @@p<0.01 vs. Hemin + rh-SESN2 group; &&p<0.01 vs. Hemin + rh-SESN2 + Compound group.** |
| --- |
