# Supplementary material for: Sestrin2 Mitigates Neuronal Ferroptosis Following Subarachnoid Hemorrhage via Orchestration of the AMPK/PGC1α/Nrf2 Signaling Axis
Source: CNS Neurosci Ther. 2026 Apr 30;32(5):e70908. doi: 10.1002/cns.70908 (PMC13131070; doi:10.1002/cns.70908)
Supplement: Supplementary file 2 — Table S1: Sequences of AAV‐shRNAs targeting murine SESN2. Table S2: Sequences of siRNAs targeting murine SESN2 and Nrf2. [file CNS-32-e70908-s002.docx]

**Supplementary Tables**

**Table S1. Sequences of AAV-shRNAs Targeting Murine SESN2.**

| **Type** | **Name** | **Sequences (5'→3')** |
| --- | --- | --- |
| AAV-shRNA (mouse) | AAV-sh-SESN2 | GCCGCTCGTGACGTAGAAGTTCAAGAGACTTCTACGTCACGAGCGGCTTTTT |
|  | AAV-shRNA-NC | GCCGACAGCGTGTGCTAAGTTCAAGAGACTTAGCACACGCTGTCGGCTTTTT |

**Table S2. Sequences of siRNAs Targeting Murine SESN2 and Nrf2.**

| **Type** | **Name** | **Sequences (5'→3')** |
| --- | --- | --- |
| siRNA (mouse) | si-M-SESN2-1 | AAGCCGCUCGUGACGUAGAAGdTdT |
|  | si-M-SESN2-2 | AAGGUUCAUGUGAACUUGCUGdTdT |
|  | si-M-SESN2-3 | AAGCUCUGAUGGAACGCAUGAdTdT |
|  | si-M-SESN2-NC | AAGCACGUCUCCGGUGAGAAGdTdT |
|  | si-M-Nrf2-1 | AAGCTGGAGAACATTGTCGAGdTdT |
|  | si-M-Nrf2-NC | AAGCCATTGGAGTCTGAAGAGdTdT |
